# The colloidal stability of albumin-based drug delivery systems has a profound effect on tumoricidal activity

**DOI:** 10.1080/10717544.2026.2614801

**Published:** 2026-01-20

**Authors:** Guojun Xiong, Chengwei Jiang, Andreas G. Schätzlein, Ijeoma F. Uchegbu

**Affiliations:** a School of Pharmacy, University College London, London, UK; b Nanomerics Ltd., London, UK; c Wolfson College, University of Cambridge, Cambridge, UK

**Keywords:** Abraxane, denatured albumin, colloidal stability, drug delivery, protein engineering, HSA-PLA

## Abstract

Human serum albumin (HSA) has attracted significant attention in drug delivery since the approval of Abraxane in 2005. Abraxane is a nanoparticle albumin-bound paclitaxel (nab-PTX) formulation. Although HSA offers advantages such as prolonged circulation time (half-life ~19 days) and intrinsic hydrophobic pockets, the translation of other HSA-based nanomedicines remains limited. In fact, the significant differences between native and pharmaceutical HSA in protein structure and biological interactions could hinder their translational use in drug delivery. In this study, we demonstrate that pharmaceutical HSA (*α*-helix = 17%) is structurally denatured compared with native HSA (*α*-helix = 68%), leading to rapid clearance (<1 h) from the circulation and that drug loading is driven by pharmaceutical HSA’s amphiphilicity rather than by its hydrophobic pockets. Here, we revealed that Nab-PTX is composed of protein-coated PTX solid cores. These nanosystems have insufficient surface charge (*ζ* = −13.7 mV), leading to aggregation, and low colloidal stability, resulting in premature drug release upon dilution (<0.1  mg/mL). To address these shortcomings, we developed HSA-polylactic acid (HSA-PLA) nanoparticles with enhanced negative surface charge (*ζ* = −27.4 mV) and improved colloidal stability to reduce the premature release of encapsulated PTX upon dilution (<0.01  mg/mL). In tumor models, comparative pharmacokinetics, biodistribution, and efficacy studies demonstrated that HSA-PLA (PTX) nanoparticles reduce premature drug release, resulting in greater tumor exposure (129 ± 3 vs. 90 ± 12 µg·h/g, *p* < 0.01) and superior antitumor efficacy compared with Abraxane. These improvements further suggest that optimization may require only a simple modification when guided by proper theoretical principles.

## GRAPHICAL ABSTRACT

**Figure:**
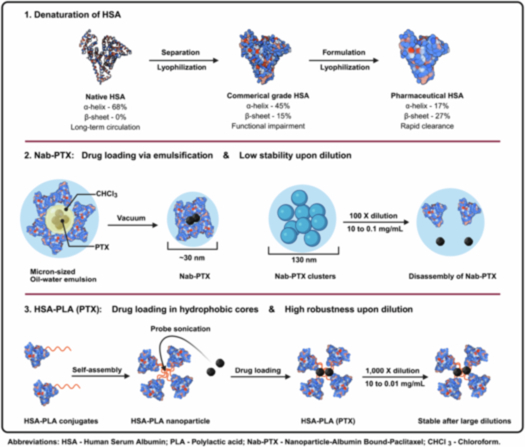

## 1. Introduction

Human serum albumin (HSA) is an endogenous, amphiphilic protein with a natural role in transporting fatty acids through the circulatory system (Curry et al. 1998). HSA has attracted increasing attention as a nanomaterial for drug delivery (Liu et al. 2024) since the approval of Abraxane by the United States Food and Drug Administration (US FDA) in 2005. Abraxane is a second-generation formulation of paclitaxel (PTX) approved for the treatment of various cancers. It is a nanomedicine composed of PTX-loaded HSA nanoparticles, also referred to as nanoparticle albumin-bound paclitaxel (Nab-PTX) (Gradishar 2006), ABI-007 and Capxol (Teng et al. 2005).

Why was only Abraxane approved as a second-generation PTX?

PTX is a cornerstone chemotherapy agent widely used as a first-line drug in the chemotherapy of multiple cancer types (Kampan et al. 2015; Mosca et al. 2021; O’Shaughnessy et al. 2021). Owing to its strong hydrophobic nature, first-generation PTX (Taxol), first approved in 1992, was formulated with Cremophor EL (a surfactant) and ethanol to disperse PTX in the aqueous medium. However, the parenteral administration of surfactants and solvents often induces severe side effects, such as fatal hypersensitivity reactions, and the Cremophor formulation limits the dose of PTX to 175 mg/m^2^, even with premedication using antiemetic and anti-allergic agents (Gradishar et al. 2005). The severe toxicity arising from this suboptimal formulation motivated the development of second-generation formulations of PTX. Therefore, at the beginning of this century, multiple surfactant- and solvent-free PTX formulations were evaluated in clinical trials (Gorelov et al. 2004; Gradishar et al. 2005; Langer et al. 2008; Xu et al. 2013; Fujiwara et al. 2019). These included PTX-polyglutamic acid conjugate (XYOTAX), tocopherol-based PTX emulsion (Tocosol), liposomal PTX (Lipusu), PTX-loaded polymeric nanoparticles (e.g. NK105), and Abraxane. Among these innovative PTX formulations, only Abraxane has demonstrated a longer time to progression (TTP) and lower toxicity compared to Taxol (Gradishar et al. 2005; Xiong et al. 2025a). In the phase 3 clinical trial comparing Abraxane and Taxol in patients with metastatic breast cancer, Nab-PTX demonstrated a longer TTP (23.0 weeks vs. 16.9 weeks, *p* = 0.006), a higher tolerable dose of PTX (260 mg/m^2^) and a significantly lower incidence of grade 4 neutropenia (9% vs. 22%, *p* < 0.001) (Gradishar et al. 2005). Grade 4 neutropenia is a key indicator of dose-limiting toxicity in chemotherapy (Ba et al. 2020). These data allowed Nab-PTX to be approved, and this medicine has now become established as the first-line formulation of PTX in chemotherapy (Schmid et al. 2020; O’Shaughnessy et al. 2021; Wang et al. 2022).

The clinical success of Abraxane has demonstrated the value of using HSA to deliver PTX (Liu et al. 2024). Therefore, tremendous efforts have been devoted to developing other HSA-based nanomedicines for cancer therapy, as well as to advancing next-generation formulations of PTX. However, in fact, in addition to Nab-PTX, which has been successfully applied to various solid tumors, only one other HSA-based formulation, Nab-rapamycin (Fyarror), has received approval from the US FDA and is used solely as an orphan drug for a rare cancer: perivascular epithelioid cell tumor (PEComa) (Liu et al. 2024). Other efforts in the clinical translation of HSA-based nanomedicines in cancer therapy, including ABI-008 (Nab-docetaxel, NCT00531271), ABI-010 (Nab-17-AAG, NCT00820768) and ABI-011 (Nab-5404, NCT01163071), were eventually terminated. In theory, as documented extensively in the literature, HSA is an endogenous protein with excellent biocompatibility and biodegradability (Li et al. 2023). It binds to the neonatal Fc receptor (FcRn), which contributes to its prolonged circulation time, and its hydrophobic pockets allow it to serve as a natural carrier for hydrophobic drugs (Cho et al. 2022). Additionally, interactions with gp60 and secreted protein acidic and rich in cysteine (SPARC) have been proposed to facilitate transendothelial transport and tumor accumulation of HSA-bound therapeutics (Hama et al. 2021). However, although HSA-bound therapeutics have promising features, their clinical translation success rate is low.

Indeed, while native HSA possesses these promising properties, pharmaceutical or commercial HSA is often partially denatured during manufacturing. This is evidenced by several studies. For example, the secondary structures of endogenous HSA are 68% *α*-helix and 0% *β*-sheet, whereas those of commercial HSA (Sigma Aldrich, UK) are 45% *α*-helix and 15% *β*-sheet. Our previous study has demonstrated that laboratory lyophilization of commercial HSA further denatured the protein, resulting in a secondary structure comprising 17% *α*-helix and 27% *β*-sheet (Xiong et al. 2025a). Consequently, PTX formulated with the denatured HSA cannot achieve prolonged circulation (Xiong et al. 2025c), even though endogenous HSA has a natural half-life of approximately 20 days (Cho et al. 2022). This rapid clearance (<1 h post-injection) of PTX has also been observed with Abraxane in both preclinical (Skoczen et al. 2020) and clinical studies (Ibrahim et al. 2002). It is clear that pharmaceutical HSA is denatured and undergoes rapid clearance in both rodents and humans, which suggests that the administration of denatured HSA may also induce an immune response (Koch et al. 1996). This may explain why the median overall survival rates observed in both the Abraxane and Taxol arms (65 weeks vs. 55.7 weeks, *p* = 0.374) are comparable, despite the higher dose of Nab-PTX compared to Taxol (260 mg/m^2^ vs. 175 mg/m^2^). Meanwhile, it has been reported that Abraxane does not bind to gp60 for transcytosis, but instead interacts with denatured albumin receptors such as gp18 and gp30 (Hama et al. 2021). Clearly, applying the properties of native HSA to pharmaceutical HSA can make data difficult to interpret, potentially causing projects to be abandoned.

The lack of awareness that pharmaceutical HSA is different from native HSA will make the development of these HSA-bound formulations difficult. In this study, in addition to demonstrating the differences between native and pharmaceutical HSA, we also aimed to reveal and clarify several facts about Abraxane, particularly regarding its drug loading, morphology, pharmacokinetics and efficacy. Under a proper theoretical framework, the issues associated with using pharmaceutical HSA in drug delivery may be clearly identified and optimized accordingly. From descriptions in their patents (Desai and Soon-Shiong 2004; Desai et al. 2008), we confirmed that Nab-PTX is prepared via an emulsification method, forming nanoparticles consisting of a PTX drug core and an HSA outer coating. This simple construction faces the challenge of disassembly under large dilutions in the bloodstream, resulting in premature drug release and thereby limiting its efficacy. Therefore, we designed an HSA-based drug delivery system featuring stronger interactions between the encapsulated drug and HSA to achieve a more robust formulation capable of resisting blood dilution. Briefly, by conjugating a biodegradable and hydrophobic polymer (polylactic acid, PLA) to the side chain of Cys34 on HSA, the resulting HSA-PLA conjugates self-assemble into core-shell structured nanocarriers, termed HSA-PLA nanoparticles (Xiong et al. 2025a). PTX can be encapsulated in the hydrophobic cores of HSA-PLA nanoparticles and protected within a stable system against blood dilution. In addition to PTX, other drugs, such as docetaxel, cabazitaxel and SN-38 can also be loaded into the hydrophobic cores of HSA-PLA nanoparticles (Xiong et al. 2025b; Xiong et al. 2025a). In the following sections, we demonstrate the cause of the low colloidal stability of Abraxane and explain how the colloidal stability of HSA nanoparticles can influence drug delivery and efficacy, supported by detailed pharmacokinetic and pharmacodynamic studies in rodents.

## 2. Methods and materials

### 2.1. Preparation of PTX-loaded HSA-PLA nanoparticles

The synthesis and drug-loading methods were identical to those described in our previous work (Xiong et al. 2025a).

***Step 1: Reduction of human serum albumin.***

HSA powder (#A1887, Sigma–Aldrich, UK, 800 mg) was dissolved in degassed sodium phosphate buffer solution (0.1 M, pH 6.8, 1 mM EDTA, 120 mL), followed by the addition of a TCEP (tris(2-carboxyethyl)phosphine) aqueous solution (1% w/v, #NB-45-00029, Generon, UK, 7 mL) to the HSA solution. The resulting mixture was stirred using a magnetic stirrer for 4 h while being placed in an ice bath and under nitrogen protection.

***Step 2: Quenching TCEP reduction.***

Dimethyl sulfoxide (DMSO, #D5879, Sigma–Aldrich, UK, 1 mL) containing 4-azidobenzoic acid (#FA35089 Biosynth Carbosynth, UK, 60 mg) was added to the reduced HSA solution. After a 10-min quenching period, degassed sodium phosphate buffer (0.2 M, pH 9.3, 40 mL) was added to adjust the reaction pH to 7. Meanwhile, the high-concentration phosphate buffer helped maintain the reaction medium at the required pH during the subsequent steps, despite the addition of DMSO.

***Step 3: Conjugating MAL-PLA to the reduced HSA.***

MAL-PLA (#746797, Sigma–Aldrich, UK, 540 mg) was dissolved in DMSO (15 mL) and subsequently added to the reduced HSA solution from above. The resulting mixture was stirred using a magnetic stirrer for 4 h at 37°C in an oil bath, all while protected with nitrogen.

***Step 4: Purification and lyophilization.***

After conjugation, the mixture underwent centrifugation using a Hermle Z232K Centrifuge (Hermle Labortechnik GmbH, Germany) for 10 min at 6000  rpm, which was repeated for 3 cycles. The supernatant was then dialyzed against distilled water in a dialysis bag (MWCO 20 K) for 2 days. Following dialysis, the dialysate was again centrifuged under the same conditions. The purified nanoparticle suspension was rapidly frozen in a −20°C solution of sodium chloride (NaCl, 25% w/v). This was followed by a 48-h lyophilization process using a freeze dryer (ALPHA 1–4 LDplus, Martin Christ, Germany). The resulting lyophilized powder was then collected and stored in a glass vial at room temperature.

PTX methanolic solution (20 mg, 1 mL, #A4393, Generon, UK) was added to the HSA-PLA nanosuspension (100 mg, 10  mL). The resulting mixtures was probe-sonicated using a Soniprep 150plus (MSE, UK) for nanoparticle formation. The sonication process consisted of two cycles, each involving 5 min of sonication at an amplitude level 5 followed by 3 min of rest, with the samples maintained in an ice bath throughout to prevent thermal degradation. After probe sonication, PTX was encapsulated into the HSA-PLA nanoparticles. Unencapsulated PTX crystals were removed through a 0.45 µm syringe filter. After a two-day lyophilization period (ALPHA 1–4 LDplus, Martin Christ, Germany), HSA-PLA (PTX) powder was stored at 4°C until further studies could be conducted. A cryoprotectant was not used in this study.

### 2.2. Preparation of PTX-loaded HSA nanoparticle with a probe sonication-based emulsification method

This method is adapted from a patented procedure for preparing Capxol, also known as Nab-PTX (Desai and Soon-Shiong 2004).

HSA was dissolved in distilled water (5% w/v, 1.8 mL, #A1653, Sigma–Aldrich, UK). Both HSA batches (A1887 and A1653) are interchangeable in this work. PTX was dissolved in chloroform (5% w/v, 0.2 mL, #A4393, Generon, UK) and then added to the HSA solution. The sonicator probe was immersed at the interface between the water and chloroform, and then, the liquid was sonicated using a Soniprep 150 Plus probe sonicator (MSE, UK) to emulsify these two immiscible phases. The sonication process consisted of two cycles, each involving 5 minutes of sonication at an amplitude level of 5 followed by 3 min of rest, with the samples maintained in an ice bath throughout. After probe sonication, the resulting emulsion was diluted with 4 mL of distilled water, and the chloroform was removed using a rotary evaporator (RV10, IKA, UK). The resulting transparent colloidal suspension was filtered through a 0.45 μm syringe filter to remove any unencapsulated PTX crystals. The filtrates were frozen at −20°C and then lyophilized (ALPHA 1–4 LDplus, Martin Christ, Germany). The resulting lyophilized powder was stored in glass vials at 4°C until further analysis could be carried out.

### 2.3. Determination of drug loading

The lyophilized PTX-loaded HSA nanoparticles (2 mg) and Abraxane (2 mg, University College London Hospital, UCLH, UK) were each dispersed in 5 mL of a 60% (v/v) acetonitrile-water solution. The resulting mixture was subjected to 2 min of vortex mixing and bath sonication. Afterward, the mixture was filtered using a 0.2 μm PTFE filter. The drug concentrations in the filtrates were determined using a high-performance liquid chromatography (HPLC) system (Agilent Technologies 1200 series, USA) equipped with a reversed-phase (RP) column (#993967-902, ZORBAX Eclipse XDB-C18, 4.6 × 150 mm, 5 µm). The mobile phase consisted of 40% water and 60% acetonitrile. The detailed parameters of this HPLC method are listed in Table 1. The HPLC chromatograms of PTX standards at different concentrations and HSA-PLA (PTX) are shown in Figure S1. In these chromatograms, the PTX elution peaks were clearly distinguished and detected at approximately 6.5 min. In practice, a 60% acetonitrile-water solution is effective for extracting PTX from HSA-based nanoparticles. This HPLC method has been previously reported (Xiong 2023).

**Table 1.** HPLC settings for the quantification of PTX.

Components	Setting
Column	#993967-902, ZORBAX Eclipse XDB-C18, 4.6 × 150 mm, 5 µm, Agilent Technologies, Inc, UK
Mobile phase	60% Acetonitrile (ACN,HPLC grade, #34851, Sigma Aldrich, UK) 40% Water (HPLC grade, #270733, Sigma Aldrich, UK)
Injection volume	10 µL
Flow rate	0.5 mL/min
Column temperature	30°C
Detection wavelength	227 nm
Runing time	10 mins
Calibration curve	y = 41691x – 9.4869
Correlation coefficient (R2)	1.0

The drug loading capacity (LC%) and encapsulation efficiency (EE%) were calculated by the following equations:

$$\mathrm{EE}\%=\frac{W_{\mathrm{encapsulated}}}{W_{\mathrm{total}}}\times 100$$

$$\mathrm{LC}\%=\frac{W_{\mathrm{encapuslated}}}{W_{\mathrm{carrier}}+\;W_{\mathrm{encapsulated}}}\times 100$$

W_encapsulated_: drug encapsulated in nanoparticles; W_total_: total drug added; W_carrier_: weight of HSA.

### 2.4. X-ray powder diffraction

The X-ray powder diffraction (XRD) patterns of the PTX standard, HSA-PLA blank nanoparticles, physical mixtures of PTX and HSA-PLA nanoparticles, PTX-loaded HSA-PLA nanoparticles, and Abraxane were determined using the following methods.

Powders were finely ground and placed on sample holders. The samples were scanned by an X-ray diffractometer (MiniFlex 600, Rigaku, Germany). The specific parameters: angle 2θ scanned from 3° to 60°, step size = 0.02°, rate of scan = 10 degree/min, X-rays (*λ* = 1.5418 Å) generated by a CuKα tube at 40 kV and 15 mA.

### 2.5. Dynamic light scattering (DLS)

The hydrodynamic diameters, zeta potentials, and polydispersity index (PDI) values of Abraxane nanoparticles, HSA-PLA blank nanoparticles, and HSA-PLA (PTX) nanoparticles in distilled water at pH 7 were determined as previously reported (Xiong et al. 2025c; Xiong et al. 2025a). PTX-loaded HSA nanoparticles were dispersed in distilled water at a concentration of 2 mg/mL, and dynamic light scattering measurements were performed using a Malvern Nano-ZS instrument (Malvern Panalytical Ltd., UK).

PTX-HSA, Abraxane and HSA-PLA (PTX) nanoparticles were also dispersed in phosphate-buffered saline (PBS, pH 7.4, #10010031, Gibco, UK) at a concentration of 2 mg/mL, and their hydrodynamic diameters and PDI values in PBS (pH 7.4) were measured using a Malvern Nano-ZS instrument. Each sample was measured in triplicate, and three independent batches were analyzed. The PBS saline was used to evaluate the hydrodynamic diameter and PDI in a physiologically relevant medium.

### 2.6. Transmission electron microscopy (TEM)

Drops of HSA-PLA (PTX), PTX-loaded HSA nanoparticles and Abraxane nanosuspension were added to TEM copper grids at a concentration of 5 mg/mL. A drop of neutral phosphotungstic acid solution (1% w/v, pH 7.0, #79690, Sigma–Aldrich, UK) was used to enhance the signal contrast of the protein nanoparticles during imaging. The prepared TEM samples were imaged at the UCL School of Pharmacy Electron Microscopy Unit.

### 2.7. Particle stability at low concentrations

#### 2.7.1. DLS measurements

The hydrodynamic diameters and PDIs of Abraxane and HSA-PLA (PTX) nanoparticles at different concentrations in phosphate buffered saline (0.001, 0.01, 0.05, 0.1, 0.3, 0.5, 1, 5, and 10 mg/mL) were measured to evaluate their particle stability at low concentrations.

#### 2.7.2. Observing tyndall effect

Phosphate-buffered saline (PBS, pH 7.4, 5 mL) and HSA saline solution (5 mL, 1 mg/mL) were used as negative controls.

Abraxane and HSA-PLA (PTX) were dispersed in PBS (pH 7.4, 5 mL) at a powder concentration of 1 mg/mL to prepare stock nanosuspensions, which served as positive controls.

Diluted samples at 0.1 mg/mL and 0.01 mg/mL were prepared from the stock suspensions for both Abraxane and HSA-PLA (PTX). The Tyndall effect was observed by directing a laser beam (R500s, Logitech, UK) through these samples in a dark environment.

### 2.8. Cell culture

The murine breast cancer cell line (4T1, CRL-2539), human breast cancer cell line (BT-549, HTB-122), human prostate cancer cell line (PC-3, CRL-1435), and human pancreatic cancer cell line (MIA PaCa-2, CRM-CRL-1420) were obtained from stocks originally purchased from ATCC (UK). The human ovarian cancer cell line (A2780) was also obtained from stocks originally purchased from Sigma-Aldrich (UK).

These cell lines were separately cultured in a tissue culture flask with a vented cap using a complete medium composed of Advanced RPMI 1640 medium (#12633012, Thermo Fisher Scientific, UK) supplemented with heat-inactivated fetal bovine serum (FBS, 1% v/v, #F9665, Sigma Aldrich, UK), GlutaMAX (1% v/v, #35050-038, Thermo Fisher Scientific, UK), and penicillin/streptomycin (1% v/v, #15140-122, Thermo Fisher Scientific, UK).

### 2.9. *In vitro* cytotoxicity

Approximately 1,000 cells of BT-549, PC-3, MIA PaCa-2, and A2780 were separately seeded into each well of 96-well plates and maintained in 100 µL of complete medium per well. These cells were incubated at 37°C in a humidified incubator with 5% CO_2_ environment, until they reached a confluence level of 50%–60%. Nab-PTX and HSA-PLA (PTX) samples were prepared and diluted in complete medium to obtain a series of predetermined concentrations. Subsequently, 100 µL of each prepared sample was added to the corresponding wells and incubated with the cells for 48 h. After treatment, the cells were washed twice with cold phosphate-buffered saline (PBS, 0.2 mL, pH 7.4), followed by the addition of 100 µL of fresh complete medium and 5 µL of WST-1 reagent (#11644807001, Roche, Sigma–Aldrich, UK) to each well for further incubation. A SPECTROstar Omega plate reader (BMG LABTECH, UK) was used to measure the absorbance of the formazan dye at 440 and 650 nm. Each experiment was performed in triplicate, and the data were analyzed using Prism software.

The cytotoxicity of HSA-PLA (PTX) nanoparticles in cell lines of BT-549, PC-3, MIA PaCa-2 and A2780 was determined in a previous study (Xiong et al. 2025a) and is adapted here for comparison.

### 2.10. Pharmacokinetics (PK) study in rats

This PK study in rats was conducted in accordance with the UK Animals (Scientific Procedures) Act 1986 and approved by the University College London Animal Welfare and Ethical Review Body (AWERB). It was carried out from April 2021 to June 2021 under the UK Home Office Project License (PP9107654) and Personal License (I1827609). The authors have adhered to the ARRIVE guidelines (https://arriveguidelines.org/).

Justification for use of animals: This PK study was designed to determine the plasma concentration‒time profile following intravenous administration of the formulation. Rats were selected as a standard and well-established model for PK evaluation of injectable drugs. The use of animals was necessary to obtain *in vivo* pharmacokinetic data, which cannot be accurately simulated by *in vitro* methods. To minimize animal use, serial blood sampling was performed via the tail vein from the same animal at multiple time points, thereby reducing the total number of rats required for the study. All procedures complied with the principles of replacement, reduction, and refinement (the 3Rs).

A total of 6 rats were used in this PK study and were handled by the author, Guojun Xiong, at the BSU of the UCL School of Pharmacy. All steps meet the requirements for personal license (No. I1827609) and project license (No. PP9107654) issued by the UK home office.

Animal housing and welfare: Rats were housed in individually ventilated cages (IVCs) in a temperature-controlled (22 ± 3°C), humidity-controlled (60 ± 5%) facility with a 12-hour light/dark cycle. The rats had ad libitum access to standard chow and filtered water. Environmental enrichment was provided, including nesting materials and shelters, was provided to reduce stress. All efforts were made to minimize animal suffering, including careful monitoring and humane endpoints.

Anesthesia: No anesthesia was required during the study, as all procedures involved were non-invasive or minimally invasive (e.g. intravenous injection), and did not cause pain or distress to the animals.

Euthanasia and humane endpoints: Rats were humanely euthanized using a rising concentration of CO_2_ followed by cervical dislocation, as approved under Schedule 1 of the UK Home Office regulations. The rats were monitored daily, and humane endpoints were defined as the body weight loss exceeding 15% or signs of systemic illness.

Six healthy female Sprague–Dawley (SD) rats (7–8 weeks old, weighing 220 ± 20 g; Charles River, UK) were randomly allocated into 2 groups, each consisting of 3 SD female rats (*n* = 3). The lyophilized powders of Abraxane nanoparticles and HSA-PLA (PTX) nanoparticles were dispersed in 0.9% NaCl saline to obtain a PTX concentration of 2.2 mg/mL for each formulation. Subsequently, 1 mL of the aforementioned PTX formulations was intravenously administered to the SD rats via the tail vein, resulting in an equivalent PTX dose of 10 mg/kg for each group. Blood samples were collected from the tail vein at various time points: 0.25, 0.5, 1, 2, 4, 8, 24, and 72 h post-injection. These samples were temporarily preserved in heparin-treated Eppendorf tubes. Following a 10-min centrifugation at 3000  rpm using a Bench Top Refrigerated Microlitre Centrifuge (Biofuge Fresco, Heraeus, Germany), 100  μL of plasma samples (400  μL for the 72-h time point group) were stored in a −20°C freezer until quantification could be performed using LC–MS/MS.

### 2.11. Pharmacokinetics and biodistribution study in tumor-bearing mice

This PK and biodistribution study in tumor-bearing mice was conducted in accordance with the UK Animals (Scientific Procedures) Act 1986 and approved by the University College London Animal Welfare and Ethical Review Body (AWERB). This study was carried out from August 2021 to December 2021 under the UK Home Office Project License (PP4970379) and Personal License (I1827609). The authors have adhered to the ARRIVE guidelines (https://arriveguidelines.org/).

Justification for use of animals: This experiment aimed to compare the tumor delivery efficiency and tissue distribution of the developed formulation with Abraxane. The 4T1 murine breast cancer model was used as a relevant tumor model to evaluate *in vivo* pharmacokinetics and biodistribution, which cannot be assessed *in vitro*. Tumor-bearing mice were therefore essential for obtaining meaningful comparative data. The minimum number of animals required to achieve reliable results was used, in accordance with the principles of replacement, reduction, and refinement (the 3Rs).

A total of 30 female mice were used in this experiment and were handled by the author, Guojun Xiong. All steps were compliant with the conditions of the personal license (No. I1827609) and project license (No. PP4970379) issued by the UK home office. These female mice (BALB/c, 8–10 weeks old, 20 ± 2 g; *n* = 30) were housed with 5 animals per cage and maintained by staff at the UCL School of Pharmacy Biological Services Unit.

Animal housing and welfare: All the animals were housed in individually ventilated cages (IVCs) in a temperature-controlled (22 ± 3°C), humidity-controlled (60 ± 5%) facility with a 12-h light/dark cycle. The mice had ad libitum access to standard chow and filtered water. Environmental enrichment was provided, including nesting materials and shelters, to reduce stress. All efforts were made to minimize animal suffering, including careful monitoring and humane endpoints.

Anesthesia: No anesthesia was required during the study, as all procedures involved were non-invasive or minimally invasive (e.g. subcutaneous injection, intravenous injection), and did not cause pain or distress to the animals.

Euthanasia and humane endpoints: Mice were humanely euthanized using a rising concentration of CO_2_ followed by cervical dislocation, as approved under Schedule 1 of the UK Home Office regulations. The mice were monitored daily, and humane endpoints were defined as body weight loss exceeding 15%, ulceration persisting for more than 3 days, or signs of systemic illness.

A total of 30 female BALB/c mice (8–10 weeks old, 20 ± 2 g) were subcutaneously injected with 4T1 cells (2 × 10^6^ cells in 100  μL of PBS) in the right flank. Approximately ten days after injection, the 4T1 tumor volume reached approximately 200 mm^3^. These tumor-bearing mice were then randomly divided into 10 groups, with 3 mice in each group.

The lyophilized powders of Abraxane and HSA-PLA (PTX) nanoparticles were reconstituted in 0.9% NaCl saline to a PTX concentration of 4 mg/mL. Five groups of 4T1 tumor-bearing mice were intravenously injected with Abraxane nanosuspension at a PTX dose of 20  mg/kg and killed at 1, 3, 8, 24, and 72 h post-injection. The remaining five groups of 4T1 tumor-bearing mice were intravenously administered HSA-PLA (PTX) nanosuspension at the same PTX dose and were killed at the same time points.

Blood samples were collected from the treated mice via cardiac puncture under anesthesia. Heart perfusion with 50 mL of PBS (pH 7.4) was subsequently performed to remove residual blood from the mice. After perfusion, the tumors, hearts, livers, spleens, lungs, and kidneys were extracted, rinsed with PBS, and then stored in a −20°C freezer.

Mouse blood samples were prepared using the same procedure as for rat blood samples. The frozen tissue samples were thawed using a 37°C water bath, and the organs and tumors were sectioned into smaller pieces, while vessels, ducts, ligaments, fibrous appendages, and fats within the tissues were removed. These tissue pieces were subsequently weighed and suspended in PBS saline (100 mg of tissue per mL of PBS). After 10 min of probe sonication at an amplitude of 9, the tissue pieces were homogenized to create a tissue suspension. Finally, 0.1  mL of the tissue suspension (0.4  mL for the 72-h group) was aliquoted and stored at −20°C until further MTBE liquid‒liquid extraction.

### 2.12. Methyl tertiary butyl ether (MTBE) liquid–liquid extraction

The frozen plasma or tissue suspension samples obtained from above were defrosted in a 37°C water bath. These defrosted samples were treated with 100  μL of 60% ACN solution (in distilled water) and vortexed for 2 min. Another 100  μL of ACN was subsequently added, and the mixture was vortexed for an additional 2 min for protein precipitation. Following this, a volume of 2 mL of MTBE was subsequently added to the treated tissue suspension for the first extraction, and the mixture was vortexed for 5 min. The mixture was then subjected to centrifugation at 10,000 rpm for 5 min (Hermle Z232K Centrifuge, Hermle Labortechnik GmbH, Germany). The resulting supernatant was collected and transferred to a glass vial.

For the second extraction, another 2 mL of MTBE was added to the pellet obtained from the first extraction. The resulting suspension was vortexed for 5 min and then subjected to centrifugation at 10,000  rpm for 5 min. The second supernatant was combined with the first supernatant, and then the MTBE solvent was removed using rotary evaporation. The residue was dissolved in 1 mL of ACN (0.5 mL ACN for the 72-h rat plasma samples and 0.2 mL ACN for the 72-h mouse plasma samples) through vortex and bath sonication. After dissolving the residue, 0.2 μm PTFE syringe filters were used to filter the samples. Finally, these samples were subjected to quantification of PTX using LC–MS/MS (Agilent Technologies, 6460 Triple Quad LC/MS).

### 2.13. LC–MS/MS method: quantification of PTX

In LC–MS/MS, analytes are separated by a column (stationary phase) in a liquid chromatography (LC) system, and the eluted analytes are subsequently detected using a triple quadrupole mass spectrometer (MS/MS).

The settings for the LC components are listed in Table 2.

**Table 2.** Detailed settings for the LC system used in LC–MS/MS.

Components	Setting
Column	ZORBAX C18, 4.6 × 150 mm, 3.5 µm (#959963-902, Agilent Technologies, Inc., UK)
Mobile phase	60% of Acetonitrile with 0.1% Formic Acid (v/v), Optima LC/MS Grade (#LS120-1, Fisher Scientific, UK) 40% of Water with 0.1% Formic Acid (v/v), Optima LC/MS Grade (#LS118212, Fisher Scientific, UK)
Injection volume	10 µL
Flow rate	0.5 mL/min
Column temperature	40°C
Running time	10 mins

An MS range scan was first applied to detect and identify PTX ions within the mass range of 200–900 m/z, as shown in Figure S1.A. Based on their abundance, the ion at 854 m/z was selected as the precursor ion, and the ion at 286 m/z was chosen as the major product ion for establishing the calibration curve of PTX.

The parameters for the MS/MS compartment are shown below:

Detection mode: multiple reaction monitoring (MRM); Gas Temp: 300°C; Gas flow: 5 L/min; Nebulizer: 25 psi; Sheath Gas Temp: 300°C; Sheath Gas flow: 11 L/min; Capillary positive/negative: 3500 V; Precursor ions: 854.2 and 569.2; Product ions: 286.1 and 509.2; Dwell: 250; Fragmentor: 110 V; Collision energy: 12 V; Cell accelerator: 1 V and Delat EMV: 400 V.

The LC–MS/MS chromatograms of PTX at concentrations of 10,000 ng/mL, 1,000 ng/mL, 100 ng/mL, 10 ng/mL, and 1 ng/mL acquired under MRM mode, are provided in the Figure S2. B as examples. These PTX peaks in the LC‒MS/MS chromatograms are clearly resolved and eluted at approximately 4.8 min. The resulting calibration curves of PTX in various tissues are listed in Table 3. This LC‒MS/MS method has been reported previously (Xiong 2023) and provides a wide dynamic range for detecting PTX, from below 1 ng/mL to over 10,000  ng/mL. This enables accurate quantification of PTX in tissues and plasma from 15 min to 72 h post-injection.

**Table 3.** Calibration curves for PTX determined by LC-MS/MS.

Tissues	Calibration curve	Correlation coefficient (R^2^)
Plasma	y = 223.06x − 137.59	1.0
Heart	y = 193.44x − 1792.9	1.0
Liver	y = 210.05x − 747.15	0.9999
Spleen	y = 228.88x − 316.38	0.9994
Lung	y = 215.03x − 147.39	1.0
Kidney	y = 209.28x + 367.84	1.0
Tumor	y = 229.39x + 27.64	0.9999

### 2.14. *In vivo* anticancer study

These *in vivo* anticancer studies were conducted in accordance with the UK Animals (Scientific Procedures) Act 1986 and approved by the University College London Animal Welfare and Ethical Review Body (AWERB). *In vivo* anticancer studies in 4T1 tumor models were carried out from February 2022 to April 2022, and those in MDA-MB-231 tumor models were conducted from November 2022 to February 2023. Both studies were performed under the UK Home Office Project License (PP4970379) and Personal License (I1827609). The authors have adhered to the ARRIVE guidelines (https://arriveguidelines.org/).

Justification for use of animals: animal models are essential for evaluating therapeutic efficacy in a biologically relevant setting. The use of NOD-SCID mice is justified by their immunodeficient status, which permits the engraftment and growth of human tumor xenografts without immune rejection, enabling the study of human-specific cancer biology and drug response. Immunocompetent BALB/c mice were used to evaluate the antitumor efficacy of the formulations within a fully functional immune system, providing critical insights into their translational feasibility.

A total of 30 female mice were used in this experiment and were handled by the author Guojun Xiong. All steps were compliant with the conditions of the personal license (No. I1827609) and project license (No. PP4970379) issued by the UK home office. Fifteen immunocompetent female mice (BALB/c, 8–10 weeks old, 20 ± 2 g; *n* = 15) were housed in 5 animals per cage and maintained by staff at the UCL School of Pharmacy Biological Services Unit. Fifteen immunocompromised female mice (NOD-SCID, 10 weeks old, 22 ± 2 g; *n* = 15) were housed in 5 animals per cage in the SCID room and maintained by staff at the UCL Institute of Child Health Biological Services Unit.

For animal housing and welfare, all the animals were housed in individually ventilated cages (IVCs) in a temperature-controlled (22 ± 3°C), humidity-controlled (60 ± 5%) facility with a 12-h light/dark cycle. The mice had ad libitum access to standard chow and filtered water. Environmental enrichment, including nesting materials and shelters, was provided to reduce stress. All efforts were made to minimize animal suffering, including careful monitoring and humane endpoints.

Anesthesia: No anesthesia was required during the study, as all procedures involved were non-invasive or minimally invasive (e.g. subcutaneous injection, intravenous injection), and did not cause pain or distress to the animals.

Euthanasia and humane endpoints: Mice were humanely euthanized using a rising concentration of CO_2_ followed by cervical dislocation, as approved under Schedule 1 of the UK Home Office regulations. The mice were monitored daily, and humane endpoints were defined as a tumor volume of 1200 mm^3^, body weight loss exceeding 15%, ulceration persisting for more than 3 days, or signs of systemic illness. To prevent tumor volumes from exceeding the 1200 mm^3^ humane endpoint, the animals were euthanized when the tumor size reached approximately 600–1000 mm^3^.

#### 2.14.1. 4T1 tumor model

A total of 15 female BALB/c mice (8−10 weeks old, 20 ± 2 g, *n* = 15) were subcutaneously injected with 4T1 cells (2 × 10^6^ cells in 100  μL of PBS) in the right flank. Approximately 7 days after the subcutaneous injection, the tumor volume reached approximately 100 mm^3^. These 4T1 tumor-bearing mice were randomly divided into 3 groups with 5 mice in each group (*n* = 5). The groups were as follows: the control group treated with HSA standard in saline, the Abraxane group (equivalent to 40 mg/kg PTX), and the HSA-PLA (PTX) group (equivalent to 40 mg/kg PTX). The mice received two intravenous injections on day 0 and day 3. The 4T1 tumor-bearing mice were euthanized when the tumor volume was approximately 600 mm^3^.

#### 2.14.2. MDA-MB-231 tumor model

MDA-MB-231 cells (CRM-HTB-26, ATCC, UK) were maintained in a non-filter capped tissue culture flask filled with the Leibovitz’s L-15 medium (#11415-049, Thermo Fisher Scientific, UK), supplemented with heat inactivated FBS (10% v/v) and penicillin/streptomycin (1% v/v), and incubated at 37°C in a CO_2_ free environment at 95% humidity.

A total of 15 NOD-SCID female mice (10 weeks, 22 ± 2 g, *n* = 15, Charles River Laboratories, UK) were subcutaneously injected with MDA-MB-231 cells (1 × 10^7^ in 100  μL of PBS) in the right flank. Once the tumor volumes reached approximately 100 mm^3^, the mice were randomly allocated into 3 groups (with 5 mice in each group, *n* = 5). These groups were designated as follows: untreated group, Abraxane group and HSA-PLA (PTX) group. The mice were intravenously administered with 0.1  mL of the corresponding drugs at an equivalent dose of 20  mg/kg on days 0, 3, 6, 9, and 12.

### 2.15. Statistics

Data are presented as means ± standard deviation (SD). A Student's *t*-test was employed to analyze particle size comparisons and results from in vitro cytotoxicity, pharmacokinetic, and biodistribution studies. Two-way ANOVA was applied for the statistical analysis of anticancer studies. All the statistical analyses were performed using Prism software. A *p*-value of < 0.05 was considered statistically significant.

## 3. Results and discussion

### 3.1. Drug loading of Nab-PTX via emulsification

**Figure 1.**
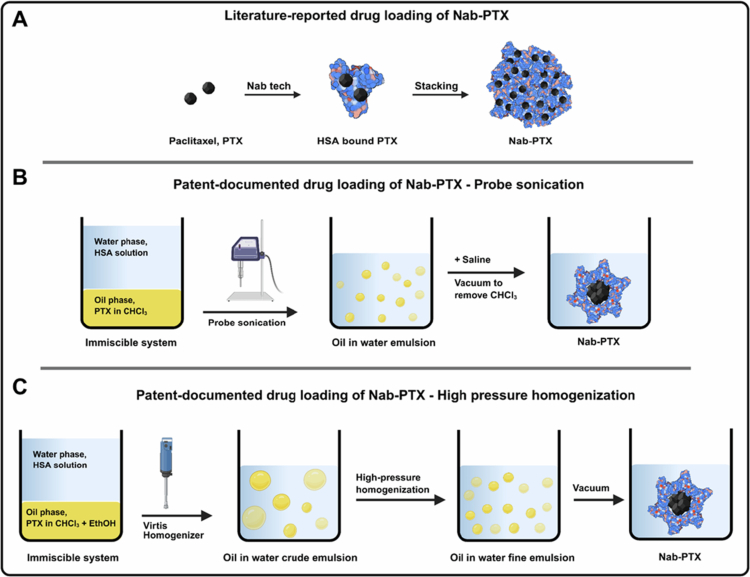
Drug loading of Abraxane nanoparticle (Nanoparticle Albumin Bound Paclitaxel, Nab-PTX). (A) Schematic illustration of the literature-reported drug-loading mechanism of Nab-PTX. PTX molecules are hypothesized to be incorporated into the hydrophobic pockets of HSA, forming albumin-bound PTX complexes that subsequently stack to form nanoparticles. (B) Drug-loading method for Nab-PTX as described in the patent (US 6,753,006 B1). The immiscible system of HSA aqueous solution and PTX in chloroform (CHCl_3_) is emulsified by probe sonication, followed by the addition of saline and vacuum evaporation to remove chloroform, resulting in nanoparticles with a protein shell and a PTX core. (C) Patent-documented drug loading of Nab-PTX via a high-pressure homogenization method (US 2008/0161382 A1). The immiscible system of HSA solution and PTX in chloroform/ethanol was first processed using a homogenizer to produce a crude oil-in-water emulsion. High-pressure homogenization refines this into a fine emulsion, and subsequent vacuum removal of organic solvents yields PTX-loaded HSA nanoparticles. Created with Biorender.com.

The drug-loading method for Nab-PTX is briefly described in its clinical publications as involving high-pressure homogenization to formulate PTX and HSA into a nanoparticle-based colloidal suspension (Ibrahim et al. 2002). The drug-loading mechanism of PTX into HSA involves the incorporation of PTX into the hydrophobic pockets of HSA (Desai 2016), leading to the stacking of albumin-bound PTX into the nanoparticles, as briefly illustrated in Figure 1A. However, the secondary structures of pharmaceutical and native HSA are significantly different (Xiong et al. 2025a). The characteristics of native HSA, such as a long circulation time and gp60 binding, do not apply to pharmaceutical HSA. It is reasonable to question whether the hydrophobic pockets of pharmaceutical HSA still function similarly to those of native HSA. Meanwhile, as the efficacy of Nab-PTX in improving patients’ overall survival rates is not significantly different from that of Taxol (Luhn et al. 2019), there is a motivation to develop next-generation PTX formulations with improved efficacy and comparable safety profiles. Optimizing Nab-PTX to enhance PTX efficacy may be a straightforward and effective strategy. Therefore, understanding the formation process of Nab-PTX, as well as the advantages and shortcomings of this drug-loading method, is vital for designing an optimized strategy.

However, the preparation process of Nab-PTX has rarely been reported in the literature. Therefore, we began searching for drug-loading methods in patent publications. Notably, in addition to ABI-007 and Nab-PTX, Abraxane also is known as Capxol (Teng et al. 2005). In the patent publications of Capxol (Desai and Soon-Shiong 2004; Desai et al. 2008), we found detailed information on the preparation of Nab-PTX. In U.S. Patent (US 6,753,006 B1), a probe sonication-based emulsification method for the preparation of Capxol is described in Example 6: *Preparation of protein-walled polymeric shells containing a solid core of a pharmaceutically active agent*. This drug loading process is briefly illustrated in Figure 1B. An HSA solution (5% w/v) was used as the aqueous phase, and PTX was dissolved in an organic solvent, such as chloroform (CHCl_3_). Under probe sonication, the immiscible phases were mixed to form a finely dispersed emulsion. Following dilution with normal saline and rotary evaporation to remove CHCl_3_, a colloidal suspension was obtained, consisting of nanoparticles with a protein-coated solid drug core.

In U.S. Patent (US 2008/0161382 A1), a high-pressure homogenization-based emulsification method for the preparation of Capxol was reported in Example 4: *Preparation of less than 200 nm sterile-filterable nanoparticles*. As illustrated in Figure 1C, the immiscible phases were first mixed using a Virtis homogenizer to create a crude emulsion. This crude emulsion was then processed with a high-pressure homogenizer to produce a finely dispersed emulsion. Following rotary evaporation to remove CHCl_3_ and ethanol, a transparent colloidal suspension was obtained.

It is now clear that the nature of Abraxane’s drug loading method (Nab technology) does not involve inserting drug molecules into the hydrophobic pockets of HSA. Instead, the emulsification-based drug-loading method, using CHCl_3_ droplets containing PTX to create temporary hydrophobic cores that are coated with HSA. Other organic solvents may also be applicable in the industrial process. This drug-loading principle relies on the amphiphilic properties of pharmaceutical HSA rather than using its hydrophobic pockets.

In this study, we reproduced PTX-loaded HSA nanoparticles using the probe sonication method described in the Capxol patent, in order to gain a better understanding of the drug-loading approach. Briefly, PTX was dissolved in CHCl_3_ at a concentration of 50  mg/mL, and HSA was dissolved in distilled water at the same concentration. The PTX solution (10 mg, 0.2 mL) was added to the HSA solution (90 mg, 1.8 mL) and emulsified to form an oil‒water emulsion. After removing CHCl_3_, PTX-loaded HSA nanoparticles were obtained. As illustrated in Table 4, the drug encapsulation efficiency and loading capacity of this method are 101.2 ± 7.5% and 10.1 ± 0.8%, respectively. For this method, a limited volume of CHCl_3_ (2 mL per gram of PTX-loaded HSA nanoparticles) was used. Meanwhile, the entire drug-loading process is very simple, making it easy to reproduce and scale up. The drug encapsulation efficiency (slightly exceeding 100%) is likely due to the high volatility of CHCl_3_, which led to inaccurate aspiration of PTX with a pipette and resulted in variability in drug loading.

**Table 4.** Physicochemical characterization of PTX formulations, including hydrodynamic diameter, polydispersity index, zeta potential, and drug loading efficiency. Data are presented as mean ± SD, *n* = 3.

	PTX-HSA NPs	Abraxane	HSA-PLA (PTX)
Hydrodynamic diameter (nm)	198 ± 31	163 ± 3	168 ± 17
PDI	0.10 ± 0.03	0.07 ± 0.02	0.08 ± 0.01
Zeta-potential (mV)	−16.7 ± 7.0	−13.7 ± 3.1	−27.4 ± 2.4
EE% (w/w)	101.2 ± 7.5	Not applicable	90.3 ± 2.6
LC% (w/w)	10.1 ± 0.8	10	15.1 ± 0.4

Drug loading via oil‒water emulsification has been extensively investigated. Owing to its amphiphilicity, HSA acts as an emulsifier to stabilize oil droplets in the water phase. With rotary evaporation of CHCl_3_, PTX becomes supersaturated in the oil droplets and gradually separates into solid cores, which are then coated and stabilized by HSA. Therefore, during this process, parameters such as HSA concentration, PTX concentration in CHCl_3_, oil‒water droplet size, and evaporation rate influence particle formation and size (Lomis et al. 2016). As exemplified in our supplemental materials (Figure S3), PTX-loaded HSA nanoparticles with LC% values of 9.7% and 11.5% exhibited significantly different particle sizes and morphologies: 16 ± 3  nm and 93 ± 82  nm, respectively, *p* < 0.0001. When the PTX loading in HSA nanoparticles exceeded 11% w/w, a significant portion of the nanoparticles appeared cracked after lyophilization (Figure S3), suggesting that a drug loading of approximately 10% w/w may be optimal in this method, which is consistent with the LC% value (10% w/w) of Abraxane. Notably, the PTX content in Abraxane was determined to be 9.5 ± 0.4% w/w, which is attributable to the addition of sodium caprylate and the *N*-acetyl-DL-tryptophan formulation (Figure S4).

Overall, Nab-PTX is prepared using an emulsification method, which offers advantages in simplicity, reproducibility, and scalability. Additionally, its drug-loading principle is based on coating HSA onto solid PTX cores, rather than incorporating PTX molecules into the hydrophobic pockets of individual HSA molecules. To avoid particle disruption after lyophilization, the PTX loading in HSA nanoparticles is suggested to be kept below 11% w/w.

### 3.2. Morphology of Nab-PTX

The reported morphology of Abraxane is a 130 nm spherical nanoparticle formed by the stacking of numerous HSA molecules, each bound with PTX (Desai 2016). However, this morphological assumption is based on the loading of PTX molecules into the hydrophobic pockets of HSA. In this work, we demonstrated that the drug loading of Abraxane is achieved through an emulsification process in which HSA serves as a coating material to stabilize the solid PTX core. Therefore, it raises the question of whether the actual morphology of Abraxane is consistent with what has been described in the literature. This is important, as the drug release mode is different according to these two morphological descriptions.

**Figure 2.**
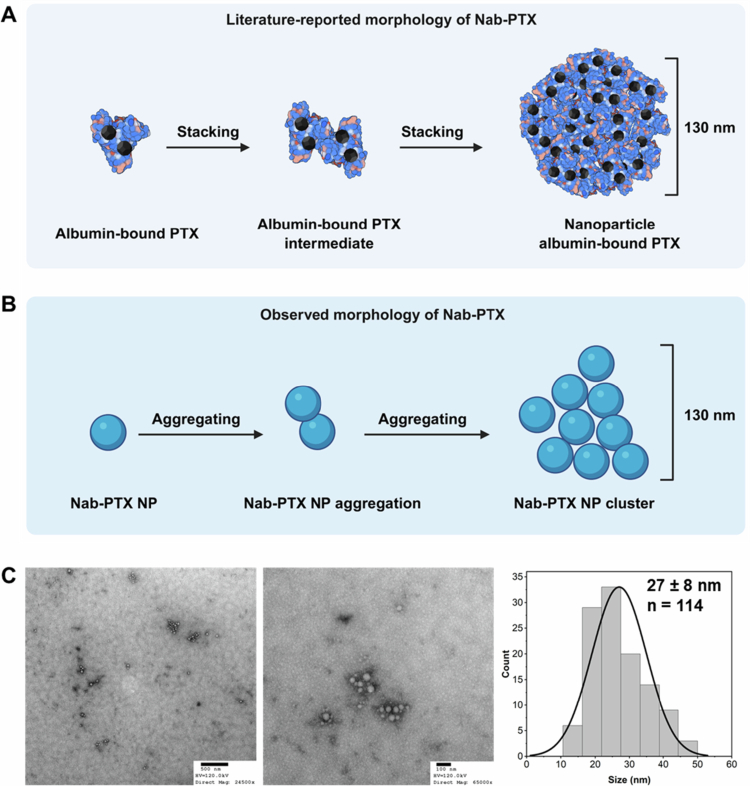
Literature-reported versus observed morphology of Nab-PTX. (A) Literature-reported morphology of Nab-PTX. Nab-PTX is hypothesized to form through the stacking of albumin-bound PTX complexes into nanoparticles with an average reported diameter of ~130 nm. Created with Biorender.com. (B) Observed morphology of Nab-PTX. Experimental observations indicate that Nab-PTX consists of smaller nanoparticles (27 ± 8 nm) that aggregate to form larger clusters of ~130  nm. Created with Biorender.com. (C) TEM images of Nab-PTX nanoparticles, along with particle size distribution analysis (*n* = 114). Left TEM image of Abraxane was adapted from our previous work (Xiong et al. 2025c).

In the TEM images of Abraxane (Figure 2), the nanoparticles clearly exhibit a spherical morphology with a diameter of 27 ± 8 nm. This small particle size corresponds with other reported TEM images of Abraxane nanoparticles, which also show spherical particles approximately 30 nm in diameter (Zhao et al. 2015). The observed morphology of Abraxane nanoparticles is significantly different from the morphology proposed in the literature, as illustrated in Figure 2. A and B, in that the final Abraxane formulation consists of aggregated small 28 nm nanoparticles all joined up to present as a 130 nm nanosystem and not a collection of HSA and PTX molecules making a 130 nm nanoparticle. Interestingly, dynamic light scattering (DLS) measurements (Table S1 in the supplementary materials) showed that the hydrodynamic diameter of Abraxane nanoparticles in phosphate-buffered saline (PBS, pH 7.4) was 128 ± 8 nm, which is close to the commonly reported diameter of 130 nm.

Why do TEM images and DLS measurements show significantly different results? This can be explained by the principle of DLS measurements, in which the hydrodynamic diameter is calculated by monitoring the Brownian motion of moving entities in suspension (Stetefeld et al. 2016). When the 28 nm Abraxane nanoparticles aggregate and move as particle clusters, the hydrodynamic diameter increases significantly. The aggregation of Abraxane nanoparticles was observed in their TEM images (see Figure 2C). The appearance of these irregularly shaped particle clusters also corresponds to that observed in cryo-EM images of Abraxane reported in other publications (Bhattacharyya et al. 2015; Callmann et al. 2019). This phenomenon was also reproduced in our prepared PTX-HSA nanoparticles, where numerous tiny PTX-HSA nanoparticles aggregated into large clusters (Figure S3), resulting in hydrodynamic diameters that were significantly larger than their particle sizes (198 nm vs 16 nm).

In fact, the aggregation of nanoparticles is a common phenomenon. According to the Derjaguin–Landau–Verwey–Overbeek (DLVO) theory, if the electrostatic repulsion between particles is not sufficiently strong, the van der Waals attractive forces will dominate, leading to particle aggregation to minimize the system's free surface energy (Adair et al. 2001). In order to quantitatively represent the magnitude of repulsive forces in colloidal systems, the absolute value of the zeta potential of nanoparticles serves as a key parameter for assessing both interparticle repulsion and colloidal stability (Hunter 2013). The measured zeta potential of Abraxane in distilled water is −13.4 ± 3.7 mV, which is not sufficiently high to ensure colloidal stability, thereby leading to particle aggregation into clusters. This may explain the significant discrepancy between the reported and our observed particle sizes of Abraxane nanoparticles (130 nm vs. 27 nm).

Both the findings on the drug-loading mechanism and the particle aggregation behavior of Abraxane nanoparticles suggest that the official descriptions of Abraxane in terms of drug loading and morphology are inappropriate. As a result, the description of Abraxane’s drug release mode in which PTX molecules loaded on albumin molecules gradually dissociate from Abraxane nanoparticles upon large dilution in the bloodstream may be inaccurate.

### 3.3. Poor colloidal stability of Nab-PTX upon large dilution

Colloidal stability, especially at low concentrations, is important in nanomedicine for maintaining therapeutic efficacy and altering the biodistribution of the active ingredient. Once Abraxane nanoparticles (1 g of nanoparticles) are injected into the human bloodstream (average blood volume ≈ 5 L) (Feher 2012), they undergo significant dilution during circulation at an estimated nanoparticle concentration of 0.2  mg/mL in the blood. If the colloidal system is not sufficiently robust, the particles may disassemble and release the drug prematurely into the bloodstream.

**Figure 3.**
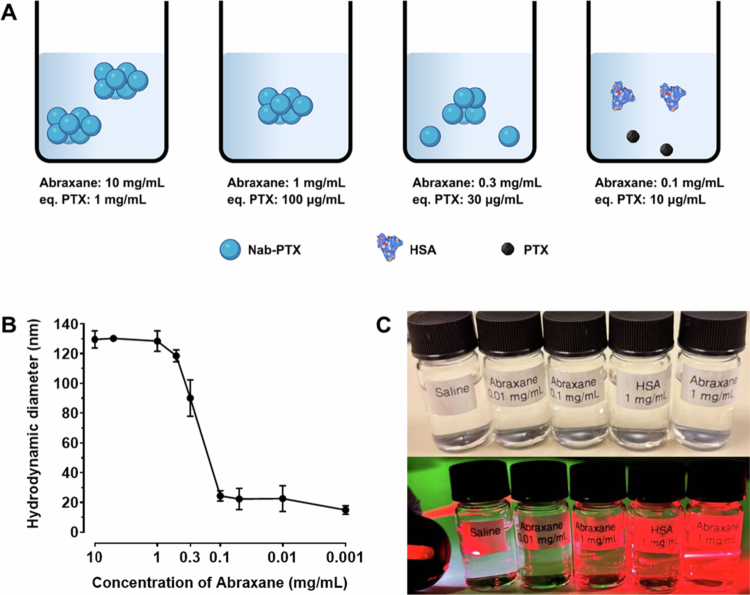
Colloidal stability of Abraxane under dilution conditions. (A, B) Schematic illustration of Abraxane stability at different concentrations. Abraxane nanoparticles are stable at concentrations of 10 and 1 mg/mL. Upon dilution to 0.3  mg/mL, partial disassembly occurs, and at 0.1  mg/mL, the Abraxane nanoparticles fully disassembled into free HSA and PTX molecules. Created with Biorender.com. (C) Examination of the Tyndall effect of Abraxane at different concentrations. The visible laser path (Tyndall effect) confirmed the colloidal state of Abraxane at 1  mg/mL, whereas the effect disappeared at 0.1  mg/mL, indicating the loss of colloidal nanoparticles.

In this study, we tested the robustness of Abraxane nanoparticles when they were subjected to large dilutions by monitoring their hydrodynamic diameters at different concentrations in saline. This method was adapted from the report *‘Novel Method to Determine the Bioequivalence of Nanomedicine’* by the Nanotechnology Characterization Laboratory (NCL) of the National Cancer Institute (NCI). As illustrated in Figure 3A and B, Abraxane nanoparticles were stable when diluted from 10 to 1 mg/mL. However, upon further dilution to 0.1  mg/mL, the nanoparticles disassembled into free albumin molecules. This disassembly process was also visualized by the Tyndall effect observed at different concentrations, as shown in Figure 3C. The light path of the red laser should be visible in the colloidal solution because of the Tyndall effect (Lei et al. 2015). It is clear that Abraxane does not form a colloidal system at a concentration of 0.1 mg/mL, indicating that the nanoparticles rapidly disassemble into albumin molecules when diluted from 1 to 0.1 mg/mL. As discussed in the previous sections, Nab-PTX is a nanoparticle consisting of a solid PTX core and an HSA shell, rather than PTX being loaded into the hydrophobic pockets of HSA. Therefore, upon large dilutions, HSA redissolves into the aqueous medium, and the PTX in the inner core is subsequently released. This result may explain why NCL reported that more than 90% of PTX was unencapsulated in rat plasma following intravenous injection of Abraxane (Skoczen et al. 2020).

Therefore, we hypothesize that the low robustness of Abraxane upon large dilution may result in premature drug release, thereby reducing drug delivery efficiency.

### 3.4. Proposed strategy for improving colloidal stability – HSA-PLA (PTX)

Based on the above discussion, Nab-PTX is prepared using an emulsification method in which nanosized CHCl_3_ droplets containing PTX serve as temporary hydrophobic vehicles, and these suspended PTX-CHCl_3_ droplets are stabilized and coated with HSA in aqueous medium through HSA’s amphiphilic properties, ultimately obtaining protein-walled, PTX-loaded HSA nanoparticles after CHCl_3_ removal. This drug-loading method coats HSA onto a nanosized PTX solid core using a minimal amount of organic solvent without requiring chemical synthesis or modification, which is advantageous for scale-up production and clinical translation. However, this unstable packaging also makes it less robust when encountering large dilution in the bloodstream, potentially leading to premature drug release. Clinical evidence also reveals that the efficacy of Nab-PTX is comparable to that of Taxol, even when a higher dose of PTX is administered (Luhn et al. 2019).

**Figure 4.**
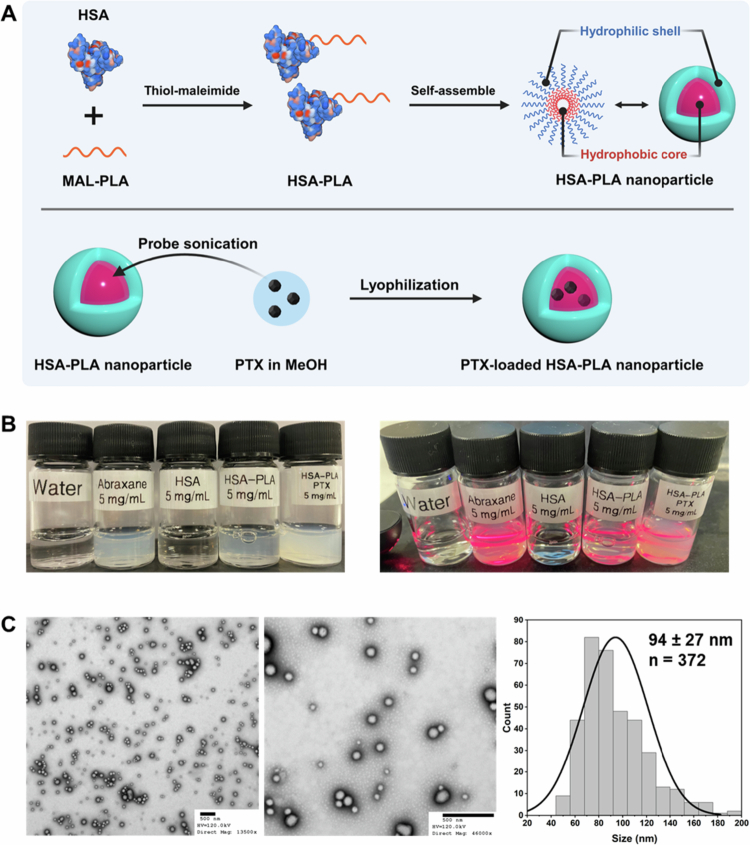
Preparation and characterization of HSA-PLA (PTX) nanoparticles. (A) Schematic illustration of HSA-PLA nanoparticle preparation. HSA is conjugated with maleimide-PLA (MAL-PLA) via thiol-maleimide chemistry, followed by self-assembly into HSA-PLA nanoparticles with a hydrophobic core and hydrophilic shell. PTX is encapsulated by probe sonication and lyophilization, yielding PTX-loaded HSA-PLA nanoparticles. Created with Biorender.com. (B) Photographs of water, Abraxane (5 mg/mL), HSA (5 mg/mL), HSA-PLA (5 mg/mL), and HSA-PLA (PTX) (5 mg/mL) liquids under ambient light (left) and illuminated with a red laser to visualize the Tyndall effect (right). (C) TEM images of PTX-loaded HSA-PLA nanoparticles and particle size distribution analysis, showing an average particle size of 94 ± 27 nm (*n* = 372). Right TEM image of HSA-PLA (PTX) nanoparticle was adapted from our previous work (Xiong et al. 2025c).

Therefore, in this work, we aimed to use a more stable HSA-based delivery system to increase the robustness of the PTX-loaded nanosystem upon large dilution, thereby enhancing drug delivery to tumors and improving therapeutic efficacy. Briefly, we conjugated maleimide-polylactic acid (MAL-PLA) to the thiol group of Cys34 on HSA, and the resulting HSA-PLA conjugates self-assembled into HSA-PLA nanoparticles in the aqueous medium, as illustrated in Figure 4A. Notably, Cys34 of native HSA has a free thiol group, whereas the thiol group of Cys34 in commercial HSA is largely blocked. This finding was systematically demonstrated in our previous work (Xiong et al. 2025a). Therefore, the reduction of HSA with TCEP is necessary before conjugating the MAL-PLA to HSA. Self-assembled HSA-PLA forms polymeric micelles with a core‒shell nanoparticle structure, where the hydrophobic core is used for efficient loading of hydrophobic drugs, and the hydrophilic shell enables colloidal stability in aqueous environments. Notably, positively charged amino acids are enriched around Cys34 and may also be incorporated into the cores of HSA-PLA nanoparticles during the self-assembly process (Xiong et al. 2025a). This makes the surface charge of HSA-PLA nanoparticles significantly more negative than that of both HSA and Nab-PTX (−26.3 ± 0.5 mV vs. −12.6 ± 3.3 mV (HSA), *p* < 0.01; −26.3 ± 0.5 mV vs. −13.4 ± 3.7 mV (Nab-PTX), *p* < 0.01). Encapsulating PTX within the cores of HSA-PLA nanoparticles does not significantly alter the surface charge of the HSA-PLA NPs, with values of −27.4 ± 2.4 vs. −26.3 ± 0.5 mV, *p* = ns. As shown in Figure 4B, the Tyndall effect was observed in the nanosuspensions of HSA-PLA blank nanoparticles, Abraxane nanoparticles, and HSA-PLA (PTX) nanoparticles at a concentration of 5  mg/mL. The XRD patterns (Figure S5) of these nanoparticles are also amorphous, further confirming that PTX is encapsulated in both Abraxane and HSA-PLA (PTX) nanoparticles. The LC% of HSA-PLA (PTX) nanoparticles was 15.1 ± 0.4%, which was higher than the 10% loading of Abraxane. No particle cracking was observed in the TEM images of the HSA-PLA (PTX) nanoparticles (see Figure 4C). In these TEM images, particle agglomeration is also observed in HSA-PLA (PTX) nanoparticles, but it is reduced compared to that of Abraxane nanoparticles. A detailed characterization of HSA-PLA (PTX) nanoparticles has been reported in our previous publication (Xiong et al. 2025a).

**Figure 5.**
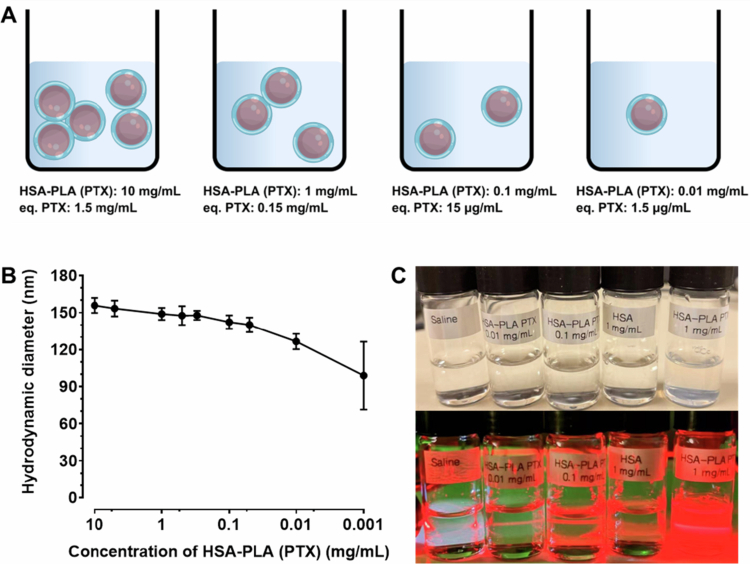
Colloidal stability and Tyndall effect of HSA-PLA (PTX) nanoparticles upon dilution. (A, B) Schematic illustration of HSA-PLA (PTX) nanoparticle stability at different concentrations (10, 1, 0.1, and 0.01 mg/mL). The nanoparticles were detectable even at 0.01  mg/mL. Cartoon created with Biorender.com. (C) Photographs of saline, HSA-PLA (PTX) (0.01 and 0.1 mg/mL), HSA (1 mg/mL), and HSA-PLA (PTX) (1 mg/mL) under ambient light (top) and under red laser illumination (bottom), demonstrating the Tyndall effect and confirming nanoparticle stability even at 0.01 mg/mL.

The critical micelle concentration (CMC) of the HSA-PLA nanoparticles was determined to be 0.5  µM (0.037  mg/mL) in our previous work (Xiong et al. 2025a). As shown in Figure S6, an aggregation-induced emission (AIE) effect of pyrene was observed with increasing concentrations of the HSA-PLA polymer in the aqueous medium. The red-shift and increased intensity of pyrene emission indicated the process of encapsulating pyrene in the HSA-PLA nanoparticles. This phenomenon has been explained by Tang and co-workers (Wang et al. 2023; Yan et al. 2025), who reported that the emission of aggregated pyrene may be enhanced due to the restriction of intramolecular motion, also known as the AIE effect (Zhao et al. 2020). Based on this AIE theoretical framework, we may conclude that the HSA-PLA conjugates start to encapsulate the hydrophobic molecules (such as pyrene) at a concentration of 10 µg/mL, as illustrated in Figure S6. B. This may explain why the PTX-loaded HSA-PLA nanoparticles remain stable upon 1000-fold dilution from 10  mg/mL to 0.01  mg/mL, as shown in Figure 5A and B. Meanwhile, the visible laser path (Figure 5C) in HSA-PLA (PTX) nanosuspensions further confirms that they remain as nanoparticles even when diluted to 0.01  mg/mL, in contrast to Abraxane, which is unstable at a concentration of 0.1  mg/mL. These findings collectively demonstrate that HSA-PLA (PTX) nanoparticles are more stable upon dilution compared to Abraxane.

### 3.5. Pharmacokinetics (PK) study in mice and rats

According to the report by NCL, nearly all PTX in the bloodstream was unencapsulated after intravenous administration of Abraxane in Sprague–Dawley rats (400 g) at a dose of 6 mg/3 mL/kg (Skoczen et al. 2020). We noticed that the estimated PTX plasma concentration at time zero (C_0_) and the concentration at 15 min post-injection (Cmax) were reported to be 1.7 and 1.1  µg/mL, respectively. Indeed, the theoretical value of initial PTX concentration can be calculated based on the injected PTX dose and the estimated blood volume of the rat. The circulating blood volume of rats and mice can be estimated at approximately 64 and 72 mL/kg, respectively (Diehl et al. 2001). This means that a 400 g SD rat has an estimated circulating blood volume of 25.6  mL. The injected dose of Abraxane was 2.4 mg of PTX in 1.2 mL of saline. Therefore, the theoretical initial concentration can be estimated at approximately 90 µg/mL, which is significantly higher than the reported C_0_ value of 1.7 µg/mL.

What causes such a significant difference?

This is a rarely discussed point in the explanation of nanomedicine PK in rodents. In fact, the heart rates of mice exceed 310 beats per minute (bpm), and those of rats are above 250 bpm (Milani-Nejad and Janssen 2014). The high heart rates also result in a large cardiac output per minute in both mice and rats. Cardiac output is estimated at approximately 214 mL/min/kg (Sweet et al. 1987) for rats and 13.5  mL/min for mice (Franco et al. 1999). These statistics indicate that mice need only a few seconds to complete full systemic blood circulation, and that rats require approximately 20–30 s. Therefore, by the time Cmax was measured at 15 min post-injection, the injected Abraxane nanoparticles had already undergone multiple systemic circulation and distribution events. Owing to the rapid clearance of Nab-PTX from the bloodstream, only a small fraction was detectable even 15 min after injection. This explains why the reported Cmax value was only 1.1 µg/mL, despite a theoretically injected concentration of 90 µg/mL. Moreover, Nab-PTX is unstable at concentrations less than 0.1 mg/mL (equivalent to 10 µg/mL PTX). As a result, most of the PTX detected in the previous report was unencapsulated. The PK of unencapsulated PTX cannot reflect the actual behavior of Nab-PTX. The key information we can conclude from this earlier report is that Nab-PTX underwent rapid clearance from the bloodstream within 15 min, and the remaining fraction was unstable in circulation.

**Figure 6.**
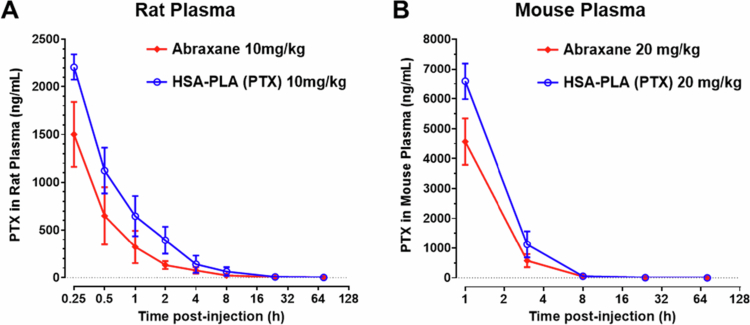
Pharmacokinetic profiles of HSA-PLA (PTX) and Abraxane in rats and mice. (A) Plasma concentration‒time curves of PTX in rats following intravenous injection of HSA-PLA (PTX) nanoparticles or Abraxane at a PTX-equivalent dose of 10 mg/kg. Adapted from our previous work (Xiong et al. 2025c). (B) Plasma concentration‒time curves of PTX in mice following intravenous injection of the same formulations at a PTX dose of 20  mg/kg. Data are presented as mean ± SD (*n* = 3 per group).

Therefore, in this study, we increased the injection concentration of Abraxane in SD rats (220 g) to 10 mg/kg, corresponding to 2.2 mg of PTX in 1 mL of saline per rat. The circulating blood volume of a 220 g SD rat is estimated to be approximately 14 mL. Therefore, the theoretical initial concentration is estimated to be approximately 150 µg/mL. However, as shown in Table 5, the Cmax of Nab-PTX in rats was determined to be 1.5 ± 0.3  µg/mL, which suggests that it was also unencapsulated. Notably, the injected HSA-PLA (PTX) exhibited significantly higher Cmax values (2.2 ± 0.1  µg/mL, *p* < 0.05) and a trend towards a greater area under the curve (AUC) compared to Abraxane (4.0 ± 1.0 µg*h/mL vs. 2.2 ± 0.5 µg*h/mL, respectively, *p* = 0.049), as shown in Table 5 and Figure 6A. These improvements are attributed to the enhanced colloidal stability of HSA-PLA (PTX) nanoparticles at concentrations below 0.01 mg/mL (equivalent to 1.5 µg/mL of PTX). The remaining PK parameters are no longer meaningful for comparison, given that the PTX of both Abraxane and HSA-PLA (PTX) in rat plasma was unencapsulated at other time points.

**Table 5.** Pharmacokinetic parameters of Abraxane and HSA-PLA (PTX) following intravenous administration in rats at a PTX dose of 10 mg/kg. Data are presented as mean ± SD (*n* = 3). Statistical significance was determined using Student’s *t*-test. A *p*-value of < 0.05 was considered statistically significant.

PK parameters	Units	Abraxane	HSA-PLA (PTX)	*p-*Value
Terminal t_1/2_	h	14.3 ± 0.5	11.8 ± 3.7	0.3107
T_max_	h	0.25	0.25	–
C_max_	µg/mL	1.5 ± 0.3	2.2 ± 0.1	0.0186
C_0_	µg/mL	3.9 ± 2.1	4.6 ± 1.7	0.6769
AUC_0-72h_	µg*h/mL	2.2 ± 0.5	4.0 ± 1.0	0.0494
AUC_∞_	µg*h/mL	2.2 ± 0.5	4.0 ± 1.0	0.0494
V_d_	L/kg	3.0 ± 1.5	2.4 ± 0.7	0.5642
CL	L*h/kg	4.8 ± 1.2	2.6 ± 0.7	0.0518

Consequently, we conducted another PK study in 4T1 tumor-bearing mice with an increased PTX dose of 20 mg/kg. This theoretical initial concentration is estimated to be approximately 270 µg/mL. The blood collection method for mice is based on cardiac puncture under anesthesia, which is more complex than tail vein sampling in rats. Therefore, the first time point was set at 1 h post-injection, as illustrated in Figure 6B. A rapid clearance of Abraxane nanoparticles and HSA-PLA (PTX) nanoparticles from the plasma of mice was also observed. As shown in Table 6, the Cmax of HSA-PLA (PTX) in mice was also significantly higher than that of Abraxane, at 6.6 ± 0.6  µg/mL vs. 4.6 ± 0.8  µg/mL, *p* < 0.05. The AUC value of HSA-PLA (PTX) was determined to be 23.0 ± 1.7 µg*h/mL, which is significantly higher than that of Abraxane, 16.1 ± 2.5 µg*h/mL, *p* < 0.05. We also observed a non-linear and higher increase in both Cmax and AUC in mice compared to those in rats. This observation is consistent with results from Abraxane’s phase I clinical trial, which showed a higher and non-linear increase in the AUC with increasing doses of 200, 300, and 375 mg/m^2^ (Ibrahim et al. 2002). This evidence also supports our hypothesis that the PK of encapsulated and unencapsulated PTX differ. Therefore, at higher doses of Abraxane, the PK profile of Nab-PTX is more representative of the encapsulated formulation. At lower doses, the plasma PTX predominantly reflects the released, unencapsulated drug. This PK comparison study in mice further supports our strategy of improving the colloidal stability of HSA-based nanoparticles to reduce the premature release and clearance of PTX.

**Table 6.** Pharmacokinetic parameters of Abraxane and HSA-PLA (PTX) following intravenous administration in 4T1 tumor-bearing mice at a PTX dose of 20 mg/kg. Data are presented as mean ± SD (*n* = 3). Statistical significance was determined using Student’s *t*-test. A *p*-value of < 0.05 was considered statistically significant.

PK parameters	Units	Abraxane	HSA-PLA (PTX)	*p-*Value
Terminal t_1/2_	h	7.8 ± 0.3	14.8 ± 2.9	0.0142
T_max_	h	1.0	1.0	–
C_max_	µg/mL	4.6 ± 0.8	6.6 ± 0.6	0.0257
C_0_	µg/mL	13.2 ± 2.6	16.5 ± 2.7	0.2020
AUC_0-72h_	µg*h/mL	16.1 ± 2.5	23.0 ± 1.7	0.0168
AUC_∞_	µg*h/mL	16.1 ± 2.5	23.1 ± 1.7	0.0168
V_d_	L/kg	1.6 ± 0.3	1.2 ± 0.2	0.1270
CL	L*h/kg	1.3 ± 0.2	0.9 ± 0.1	0.0363

### 3.6. Biodistribution study in 4T1-tumor bearing mice

The above PK results indicate that HSA-PLA (PTX) nanoparticles are more stable than Nab-PTX in terms of resistance to blood dilution. This suggests that PTX encapsulated in HSA-PLA nanoparticles has a longer blood residence time and is more readily distributed to the target tumor tissue.

In this study, we also conducted a dynamic biodistribution study to compare the distribution of Abraxane and HSA-PLA (PTX) nanoparticles in mice major organs and tumors after intravenous injection of 20 mg/kg PTX at 1, 3, 8, 24, and 72 h. In order to accurately measure PTX concentrations in organs and tumors, heart perfusion was conducted prior to tissue extraction to remove residual blood, as circulating blood also contains PTX. The appearance of organs with and without heart perfusion differs significantly; details can be found in the supplementary materials (Figure S7).

**Figure 7.**
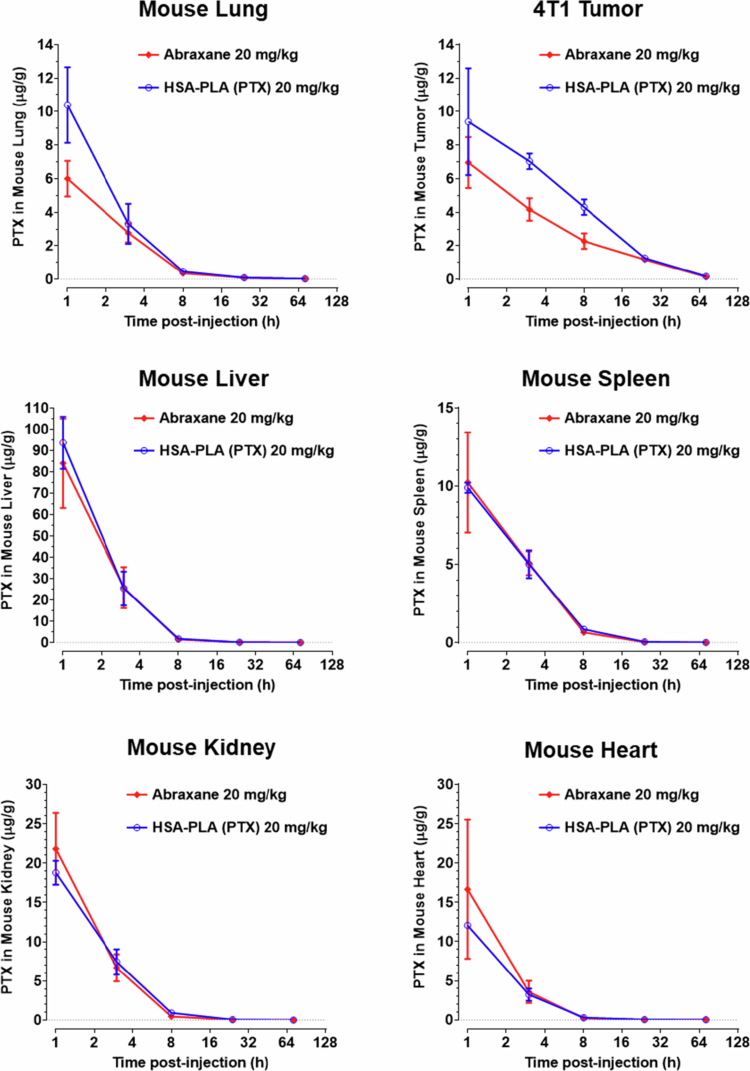
Tissue distribution of PTX following intravenous administration of HSA-PLA (PTX) nanoparticles or Abraxane in 4T1 tumor-bearing mice. PTX concentrations were measured in mouse liver, spleen, kidney, lung, heart, and tumor at various time points after intravenous injection of HSA-PLA (PTX) or Abraxane at a PTX-equivalent dose of 20  mg/kg. Data are presented as mean ± SD (*n* = 3 per group).

As illustrated in Figure 7, the rapid clearance of Abraxane and HSA-PLA (PTX) from plasma can thereby be explained, as a significant portion of the injected PTX was detected in mice liver. PTX was also detected in other major organs (hearts, spleens, lungs and kidneys) as well as in 4T1 tumors. By calculating the PTX AUC for each organ and tumor, their exposure to PTX can be quantified and compared. As shown in Table 7, the AUC value of HSA-PLA (PTX) in tumors is significantly higher than that of Abraxane (129 ± 3 µg*h/g vs. 90 ± 12 µg*h/g, *p* < 0.01), indicating an improved PTX delivery efficiency by the HSA-PLA NPs. This finding supports our hypothesis that improving the colloidal stability of HSA-based nanoparticles can reduce the premature release of PTX in plasma, thereby enhancing tumor delivery efficiency.

**Table 7.** Tissue distribution of PTX in 4T1 tumor-bearing mice following intravenous administration of Abraxane or HSA-PLA (PTX) at a PTX dose of 20 mg/kg. AUC_0-72h_ values represent the area under the concentration-time curve from 0 to 72 h in various tissues. Data are presented as mean ± SD (*n* = 3). Statistical significance was determined using Student’s *t*-test. A *p*-value of < 0.05 was considered statistically significant.

AUC_0-72h_	Unit	Abraxane	HSA-PLA (PTX)	*p-*Value
Tumor	µg*h/g	90 ± 12	129 ± 3	0.0055
Lung		26 ± 3	36 ± 2	0.0086
Liver		238 ± 55	255 ± 13	0.6299
Spleen		41 ± 6	43 ± 3	0.6328
Kidney		63 ± 10	67 ± 8	0.6172
Heart		42 ± 12	34 ± 3	0.3253

In this work, we did not exploit strategies aimed at largely prolonging the circulation time of nanosystems, as several phase 3 clinical trials have shown that extended circulation does not necessarily translate into greater survival benefits for cancer patients (O’Brien et al. 2004; Langer et al. 2008; Fujiwara et al. 2019). For example, a synthetic polymer-PTX conjugate (XYOTAX) showed significantly prolonged the circulation of PTX; however, its efficacy was even worse than that of Taxol in patients (Xiong et al. 2025a). In addition, a polymeric micelle-encapsulated PTX formulation (NK105) offers advantages such as higher drug loading and prolonged circulation time (Hamaguchi et al. 2007); however, its efficacy is also not superior to that of Taxol (Xiong et al. 2025a). Therefore, we aimed to improve the colloidal stability of the HSA-based system to enhance PTX delivery efficiency, as other synthetic polymer-based platforms have failed to achieve clinical progress, even when Compared to a PTX formulation (Taxol) developed in 1992.

The distribution of HSA-PLA (PTX) nanoparticles and Abraxane nanoparticles in the hearts, livers, spleens, and kidneys of mice are comparable. Notably, the distribution of HSA-PLA (PTX) nanoparticles in the lungs was significantly greater than that of Abraxane nanoparticles, 36 ± 2 µg*h/g vs. 26 ± 3 µg*h/g, *p* < 0.01. The improved delivery of PTX to the lungs is critical not only because of the high incidence of lung cancer, but also because the lungs represent a major site for distant metastasis (Eckhardt et al. 2012). The underlying mechanism for enhanced delivery to the lungs remains unclear. De Jong et al. demonstrated that intravenous injection of 50-nm gold nanoparticles (AuNPs) resulted in significantly higher lung deposition compared with 10-nm AuNPs (De Jong et al. 2008). We therefore hypothesize that the larger particle size of HSA-PLA (PTX) nanoparticles may contribute to their increased lung accumulation compared to Abraxane, at 94 ± 27 nm vs. 27 ± 8 nm, *p* < 0.0001. In future work, we plan to coat HSA on gold nanoparticles with different particle sizes to visualize the influence of particle size on the distribution of HSA nanoparticles.

In addition to the improved delivery of HSA-PLA (PTX) nanoparticles to 4T1 tumors, we also observed the enhanced permeability and retention (EPR) effect of PTX at 24 and 72 h post-injection, as illustrated in Figure S8 of the supplementary materials. However, the EPR effect is now considered to be heterogeneous among patients (Prabhakar et al. 2013). The heterogeneity of the EPR effect has been used to explain why drug delivery systems with prolonged circulation time do not improve drug efficacy. For example, pegylated liposomal doxorubicin exhibits a significantly longer circulation time compared to free doxorubicin (half-life: 73.9 h vs. 10 min), yet their efficacy remains comparable (O’Brien et al. 2004). Therefore, a long circulation time is not a significant criterion for evaluating the effectiveness of nanomedicine. However, nanomedicines capable of exploiting the EPR effect may provide added benefits to patients, as the EPR effect, though heterogeneous, is still observable in certain cases (Lee et al. 2017).

Based on the above results and discussion, we can conclude that, compared to Abraxane, HSA-PLA (PTX) nanoparticles enhance PTX delivery to tumors without increasing exposure to major organs, especially the heart and kidneys. As fatal cardiotoxicity and nephrotoxicity have been reported in patients treated with Taxol (Rabah 2010; Higami et al. 2022). A comparative study between Taxol and HSA-PLA (PTX) nanoparticle will be included in our next research. These promising results from the biodistribution study encouraged us to carry out *in vivo* efficacy studies.

### 3.7. *In vitro* and *in vivo* efficacy studies

The above PK and biodistribution studies collectively demonstrate that HSA-PLA (PTX) nanoparticles exhibit superior robustness against bloodstream dilution, increasing the systemic circulations of encapsulated PTX and thus improving tumor delivery compared with Abraxane.

One of the endocytic pathways of HSA-PLA nanoparticles was identified as cancer macropinocytosis in both murine and human cancer cells in our previous reports (Xiong et al. 2025c; Xiong et al. 2025b). Cancer macropinocytosis is an important pathway for scavenging proteins and nutrient substances in cancer cells and is typically upregulated to support their metabolism and proliferation (Xu et al. 2025). This may explain why delivering PTX encapsulated in HSA-based nanoparticles is important and why the premature release of PTX reduces delivery to tumors. As HSA nanoparticles are recognized as a source of nutrients and internalized by cancer cells, the encapsulated PTX can also be taken up by these cells while free PTX cannot.

**Figure 8.**
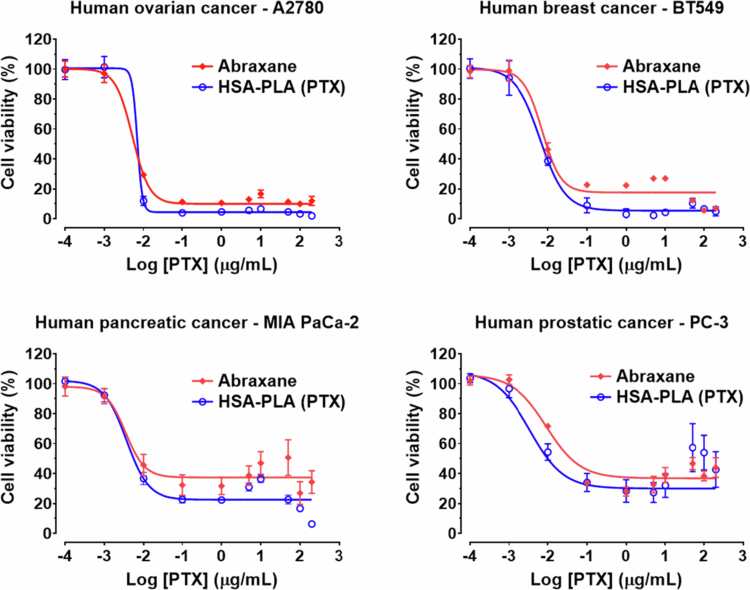
*In vitro* cytotoxicity of HSA-PLA (PTX) and Abraxane in human cancer cell lines. Data are presented as mean ± SD (*n* = 3 independent experiments per group). Data for HSA-PLA (PTX) were adapted from our previous publication (Xiong et al. 2025a).

In this study, we compared the *in vitro* cytotoxicity of HSA-PLA (PTX) and Abraxane in the A2780 (human ovarian cancer), BT549 (human breast cancer), MIA PaCa-2 (human pancreatic cancer), and PC-3 (human prostate cancer) cell lines. As listed in Table 8, both PTX formulations exhibit comparable IC_50_ values across all the tested cell lines. These IC_50_ values range from 4 to 30 ng/mL of PTX. At such low concentrations, both Abraxane and HSA-PLA (PTX) nanoparticles disassembled, resulting in unencapsulated PTX. Therefore, the IC_50_ values represent the cytotoxicity of free PTX and show no significant differences between the Abraxane and HSA-PLA (PTX) formulations. According to the cell viability-dose curves (Figure 8), at higher concentrations of PTX (0.1–10  µg/mL), HSA-PLA (PTX) nanoparticles showed greater cytotoxicity in cell lines of A2780, BT-549 and MIA PaCa-2 when compared to Abraxane. This may be due to the higher colloidal stability of HSA-PLA (PTX) nanoparticles at these concentrations, which enhances PTX delivery through encapsulation within HSA-PLA nanoparticles via the macropinocytosis pathway.

**Table 8.** IC50 values of Abraxane and HSA-PLA (PTX) in human cancer cell lines after a 48-h treatment. IC_50_ values were calculated using nonlinear regression. Data are presented as mean ± SD (*n* = 3). Statistical significance was assessed using Student’s *t*-test. A *p*-value of < 0.05 was considered statistically significant.

	Unit	Abraxane	HSA-PLA (PTX)	*p*-Value
A2780	ng/mL	6.8 ± 3.1	6.2 ± 1.9	0.7892
BT549		8.0 ± 1.6	6.5 ± 2.3	0.4063
MIA PaCa-2		6.4 ± 1.5	3.8 ± 1.4	0.0932
PC-3		26.4 ± 9.1	19.2 ± 11.2	0.4362

**Figure 9.**
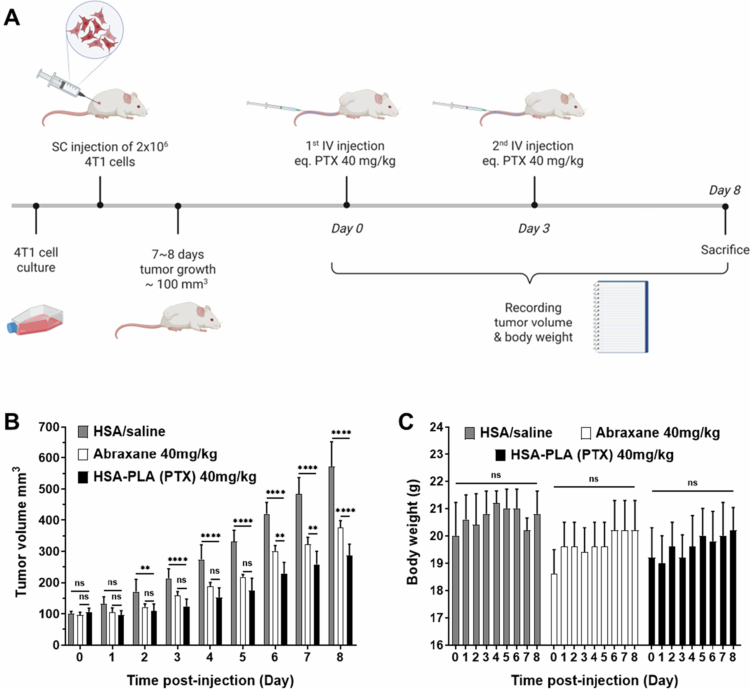
*In vivo* antitumor efficacy of HSA-PLA (PTX) nanoparticles compared with Abraxane in 4T1 tumor-bearing mice. (A) Schematic diagram of the method and treatment schedule. BALB/c mice were subcutaneously injected with 2 × 10^6^ 4T1 cells. Once the tumors reached ~100 mm^3^ (on day 0), the mice received two intravenous injections of Abraxane or HSA-PLA (PTX) at a PTX-equivalent dose of 40 mg/kg on day 0 and day 3. Created with Biorender.com. (B) Tumor volume measurements during the treatment period. (C) Body weight changes over the course of treatment. Data are presented as mean ± SD (*n* = 5 per group). **p < 0.01, ****p < 0.0001. ns = not significant.

Subsequently, we compared the *in vivo* efficacy of Abraxane and HSA-PLA (PTX) in tumor-bearing mice at the same PTX dose and dosing frequency. As illustrated in Figure 9A, 4T1 tumor-bearing mice received two injections on days 0 and 3. This 4T1 tumor model was established in immunocompetent BALB/c mice; therefore, the administration of HSA (an exogenous protein for mice) indeed induced an immune response in the mice. In practice, more than three injections of HSA may induce fatal hypersensitivity. Therefore, the dosing schedule for the 4T1 tumor model was set at two injections with a higher PTX dose (40 mg/kg). As shown in Figure 9B, mice treated with HSA-PLA (PTX) nanoparticles exhibited a significant inhibition of tumor growth when compared to control groups treated with HSA or Abraxane. Meanwhile, their body weights were not significantly changed during the treatment (Figure 9C).

**Figure 10.**
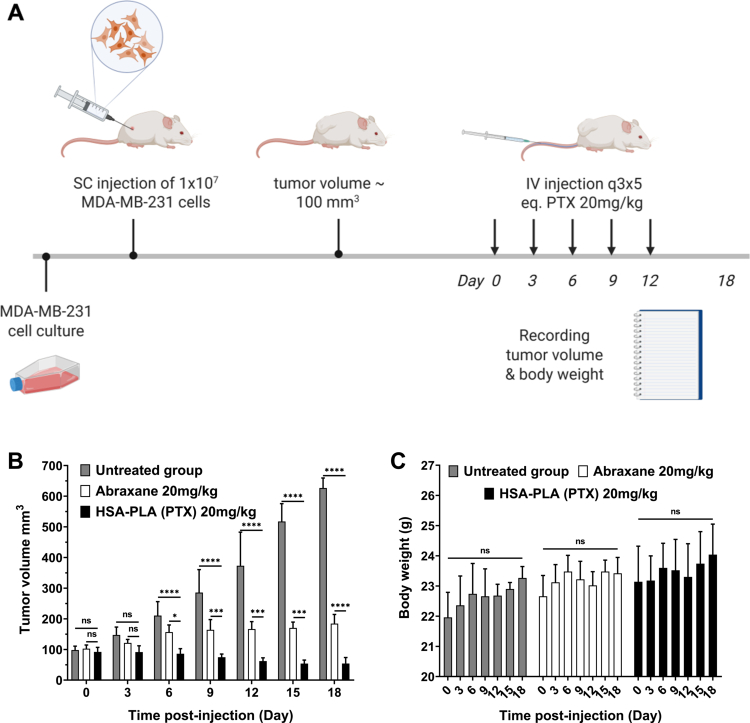
*In vivo* antitumor efficacy of HSA-PLA (PTX) nanoparticles versus Abraxane in the MDA-MB-231 xenograft model. (A) Schematic diagram of the method and treatment schedule. Female BALB/c NOD-SCID mice were subcutaneously injected with 1 × 10^7^ MDA-MB-231 cells. Once the tumor volumes reached ~100 mm^3^, the mice received intravenous injections of Abraxane or HSA-PLA (PTX) at a PTX-equivalent dose of 20  mg/kg, which was administered every three days for a total of five doses (q3 × 5). Created with Biorender.com. Adapted from the previous work (Xiong et al. 2025c). (B) Tumor volume measurements during the treatment period. (C) Body weight changes over the course of treatment. Data are presented as mean ± SD (*n* = 5 per group). *p < 0.05, ***p < 0.001. ****p < 0.0001. ns = not significant.

A humanized tumor model (MDA-MB-231) was established in immunocompromised NOD-SCID mice, which allows multiple injections without inducing hypersensitivity. As illustrated in Figure 10A, MDA-MB-231 tumor-bearing mice received 5 injections of Abraxane or HSA-PLA (PTX) on days 0, 3, 6, 9, and 12 at a PTX dose of 20 mg/kg. Mice treated with HSA-PLA (PTX) have shown superior efficacy when compared to that of Abraxane in Figure 10B. The stable body weights of the mice after treatment with HSA-PLA (PTX) nanoparticles also indicate that there were no severe side effects leading to weight loss (Figure 10C).

Based on these two *in vivo* anticancer studies, HSA-PLA (PTX) nanoparticles demonstrated enhanced efficacy in both murine and humanized tumor models in immunocompromised and immunocompetent mice, compared with Abraxane. This robust efficacy enhancement of PTX delivered by HSA-PLA nanoparticles is consistent with our PK results and suggests a reliable strategy that improves the colloidal stability of HSA-based nanoparticles to increase PTX efficacy.

## 4. Conclusion

This work aims to reveal and clarify the use of HSA as a biomaterial in the drug delivery field. The clinical success of using Nab technology to formulate PTX with HSA in nanomedicine has demonstrated the promising potential of HSA for delivering hydrophobic drugs. However, in the 20 years since, no new HSA-based nanomedicine for cancer therapy has been developed, apart from one approved for the treatment of a rare cancer. We believe that a lack of understanding of the physical chemistry of pharmaceutical HSA is one of the main reasons hindering the development of HSA-based nanomedicines. In fact, native HSA is denatured when manufactured into the pharmaceutical HSA. The inappropriate application of native HSA properties to pharmaceutical HSA will inevitably confound research expectations. A typical misconception is that PTX is incorporated into the hydrophobic pockets of the pharmaceutical HSA, whereas in fact, its loading is achieved through the amphiphilic nature of the HSA stabilizing a solid PTX core. In addition, misunderstandings about the pharmaceutical HSA have led to mischaracterization and preconceptions regarding the drug loading, morphology, and *in vivo* behavior of Abraxane. The main advantage of Abraxane is its safety, which allows a higher PTX dose compared with Taxol. The efficacy of Abraxane, even when it is administered at a higher dose, is comparable to that of Taxol in improving patients’ overall survival. These findings indicate that formulating PTX with HSA needs to be improved to enhance its efficacy. However, imprecise descriptions of the drug loading method (Nab technology) have misled strategies for optimizing HSA-based drug delivery systems. By observing the heavy agglomeration of Abraxane nanoparticles and investigating their preparation process, we conclude that the surface charge of Abraxane nanoparticles is insufficient to provide repulsive forces, and that HSA is used merely as a coating material to stabilize the PTX core. These points are very rarely mentioned and can also demonstrate the low colloidal stability of the resulting PTX-loaded HSA nanoparticles. Based on these findings, we proposed a solution by conjugating a hydrophobic polymer (PLA) to the side chain of Cys34 on HSA to drive the self-assembly of HSA-PLA into a polymeric micelle. The surface charge of HSA-PLA nanoparticles was enhanced, as more positively charged regions of HSA might be incorporated into the PLA-based cores. Meanwhile, using HSA-PLA nanoparticles to deliver PTX is more stable than using HSA itself when exposed to bloodstream dilution. Therefore, in our work, we have shown enhanced tumor delivery and tumoricidal activity with PTX-loaded HSA-PLA nanoparticles compared with Abraxane. This work clearly demonstrates the differences between native HSA and pharmaceutical HSA, as well as the advantages and limitations of Nab technology in delivering chemotherapeutics. We believe that, with a proper understanding, more effective strategies for improving HSA-based drug delivery systems will be developed and translated into clinical use.

## Supplementary Material

ARRIVE_Checklist.pdf Supplemental Material

Drug delivery supplementary materials GX.docx Drug delivery supplementary materials GX.docx

## Data Availability

Data will be made available on request from the corresponding author.

## Permission to reuse material from other sources

Permission is authorized for the adaptation of figures from our previously published open access articles entitled *Amphiphilic albumin-based nanoparticles designed for the efficient delivery of taxanes* and *Acetyl-lysine human serum albumin nanoparticles activate CD44 receptors, with preferential uptake by cancer stem cells, leading to tumor eradication*, which are distributed under the terms of the Creative Commons Attribution (CC BY) license. The reuse of these materials has been appropriately cited in the corresponding figure legends.

## References

[cit0001] Adair JH, Suvaci E, Sindel J. 2001. Surface and colloid chemistry. In: Encyclopedia of materials: science and technology. Elsevier. p. 1–10. 10.1016/B0-08-043152-6/01622-3

[cit0002] Ba Y, Shi Yuankai, Jiang W et al. 2020. Current management of chemotherapy-induced neutropenia in adults: key points and new challenges. Cancer Biol Med. 17:896–909. 10.20892/j.issn.2095-3941.2020.0069 33299642 PMC7721096

[cit0003] Bhattacharyya J, Bellucci JJ, Weitzhandler I et al. 2015. A paclitaxel-loaded recombinant polypeptide nanoparticle outperforms abraxane in multiple murine cancer models. Nat Commun. 6:7939. 10.1038/ncomms8939 26239362 PMC4753781

[cit0004] Callmann CE, LeGuyader CLM, Burton ST et al. 2019. Antitumor activity of 1,18-Octadecanedioic acid-paclitaxel complexed with human serum albumin. J Am Chem Soc. 141:11765–11769. 10.1021/jacs.9b04272 31317744 PMC6676409

[cit0005] Cho H, Jeon SI, Ahn C-H et al. 2022. Emerging albumin-binding anticancer drugs for tumor-targeted drug delivery: current understandings and clinical translation. Pharmaceutics. 14:728. 10.3390/pharmaceutics14040728 35456562 PMC9028280

[cit0006] Curry S, Mandelkow H, Brick P et al. 1998. Crystal structure of human serum albumin complexed with fatty acid reveals an asymmetric distribution of binding sites. Nat Struct Mol Biol. 5:827–835. 10.1038/1869 9731778

[cit0007] De Jong WH, Hagens WI, Krystek P et al. 2008. Particle size-dependent organ distribution of gold nanoparticles after intravenous administration. Biomaterials. 29:1912–1919. 10.1016/j.biomaterials.2007.12.037 18242692

[cit0008] Desai N. 2016. Nanoparticle albumin-bound paclitaxel (Abraxane®). In: Otagiri M, Chuang VTG, editors. Albumin in Medicine. Springer Singapore: Singapore. p 101–119. 10.1007/978-981-10-2116-9_6

[cit0009] Desai N, Soon-Shiong P. 2004. Paclitaxel-containing formulations. US 6,753,006 B1.

[cit0010] Desai N, Tao C, Soon-Shiong P. 2008. Novel formulations of pharmacological agents. Methods for the preparation thereof and methods for the use thereof. US 2008/0161382 A1.

[cit0011] Diehl K-H, Hull R, Morton D et al. 2001. A good practice guide to the administration of substances and removal of blood, including routes and volumes. J Appl Toxicol. 21:15–23. 10.1002/jat.727 11180276

[cit0012] Eckhardt BL, Francis PA, Parker BS et al. 2012. Strategies for the discovery and development of therapies for metastatic breast cancer. Nat Rev Drug Discov. 11:479–497. 10.1038/nrd2372 22653217

[cit0013] Feher J. 2012. Regulation of arterial pressure. In: Quantitative human physiology. Elsevier. p 538–548. 10.1016/B978-0-12-382163-8.00058-X

[cit0014] Franco F, Thomas GD, Giroir B et al. 1999. Magnetic resonance imaging and invasive evaluation of development of heart failure in transgenic mice with myocardial expression of tumor necrosis factor-α. Circulation. 99:448–454. 10.1161/01.CIR.99.3.448 9918534

[cit0015] Fujiwara Y, Mukai H, Saeki T et al. 2019. A multi-national, randomised, open-label, parallel, phase III non-inferiority study comparing NK105 and paclitaxel in metastatic or recurrent breast cancer patients. Br J Cancer. 120:475–480. 10.1038/s41416-019-0391-z 30745582 PMC6461876

[cit0016] Gorelov A, Gorelov S, Karlov P et al. 2004. Paclitaxel injectable emulsion: phase 2a study of weekly administration in patients with metastatic or locally advanced unresectable or recurrent urothelial transitional cell cancer (TCC). JCO. 22:4586–4586. 10.1200/jco.2004.22.90140.4586

[cit0017] Gradishar WJ. 2006. Albumin-bound paclitaxel: a next-generation taxane. Expert Opin Pharmacother. 7:1041–1053. 10.1517/14656566.7.8.1041 16722814

[cit0018] Gradishar WJ, Tjulandin S, Davidson N et al. 2005. Phase III trial of nanoparticle albumin-bound paclitaxel compared with polyethylated castor oil-based paclitaxel in women with breast cancer. JCO. 23:7794–7803. 10.1200/JCO.2005.04.937 16172456

[cit0019] Hama M, Ishima Y, Chuang VTG et al. 2021. Evidence for delivery of abraxane via a denatured-albumin transport system. ACS Appl Mater Interfaces. 13:19736–19744. 10.1021/acsami.1c03065 33881292

[cit0020] Hamaguchi T, Kato K, Yasui H et al. 2007. A phase I and pharmacokinetic study of NK105, a paclitaxel-incorporating micellar nanoparticle formulation. Br J Cancer. 97:170–176. 10.1038/sj.bjc.6603855 17595665 PMC2360299

[cit0021] Higami S, Tanaka Y, Deguchi T et al. 2022. Acute ST-segment elevations following paclitaxel administration for uterine cervical cancer: a case report and literature review. Cardio-Oncol. 8:22. 10.1186/s40959-022-00148-9 PMC9714019 36457122

[cit0022] Hunter RJ. 2013. Zeta potential in colloid science: principles and applications. Academic Press.

[cit0023] Ibrahim NK, Desai N, Legha S et al. 2002. Phase I and pharmacokinetic study of ABI-007, a cremophor-free, protein-stabilized, nanoparticle formulation of Paclitaxel1. Clin Cancer Res. 8:1038–1044. 12006516

[cit0024] Kampan NC, Madondo MT, McNally OM et al. 2015. Paclitaxel and its evolving role in the management of ovarian cancer. BioMed Res Int. 2015:413076. 10.1155/2015/413076 26137480 PMC4475536

[cit0025] Koch C, Jensen SS, Øster A et al. 1996. A comparison of the immunogenicity of the native and denatured forms of a protein. APMIS. 104:115–125. 10.1111/j.1699-0463.1996.tb00696.x 8619913

[cit0026] Langer CJ, O’Byrne KJ, Socinski MA et al. 2008. Phase III trial comparing paclitaxel poliglumex (CT-2103, PPX) in combination with carboplatin versus standard paclitaxel and carboplatin in the treatment of PS 2 patients with chemotherapy-Naïve advanced non-small cell lung cancer. J Thorac Oncol. 3:623–630. 10.1097/JTO.0b013e3181753b4b 18520802

[cit0027] Lee H, Shields AF, Siegel BA et al. 2017. 64Cu-MM-302 positron emission tomography quantifies variability of enhanced permeability and retention of nanoparticles in relation to treatment response in patients with metastatic breast cancer. Clin Cancer Res. 23:4190–4202. 10.1158/1078-0432.CCR-16-3193 28298546 PMC6790129

[cit0028] Lei W, Mochalin VN, Liu D et al. 2015. Boron nitride colloidal solutions, ultralight aerogels and freestanding membranes through one-step exfoliation and functionalization. Nat Commun. 6:8849. 10.1038/ncomms9849 26611437 PMC4674780

[cit0029] Li C, Zhang D, Pan Y et al. 2023. Human serum albumin based nanodrug delivery systems: recent advances and future perspective. Polymers (Basel). 15:3354. 10.3390/polym15163354 37631411 PMC10459149

[cit0030] Liu Y, Li Y, Shen W et al. 2024. Trend of albumin nanoparticles in oncology: a bibliometric analysis of research progress and prospects. Front Pharmacol. 15, 10.3389/fphar.2024.1409163 PMC11272567 39070787

[cit0031] Lomis N, Westfall S, Farahdel L et al. 2016. Human serum albumin nanoparticles for use in cancer drug delivery: process optimization and in vitro characterization. Nanomaterials (Basel). 6:116. 10.3390/nano6060116 28335244 PMC5302621

[cit0032] Luhn P, Chui SY, Hsieh “Angela” Fu-Chi et al. 2019. Comparative effectiveness of first-line nab-paclitaxel versus paclitaxel monotherapy in triple-negative breast cancer. J Comp Eff Res. 8:1173–1185. 10.2217/cer-2019-0077 31394922

[cit0033] Milani-Nejad N, Janssen PML. 2014. Small and large animal models in cardiac contraction research: advantages and disadvantages. Pharmacol Ther. 141:235–249. 10.1016/j.pharmthera.2013.10.007 24140081 PMC3947198

[cit0034] Mosca L, Ilari A, Fazi F et al. 2021. Taxanes in cancer treatment: activity, chemoresistance and its overcoming. Drug Resist Updates. 54:100742. 10.1016/j.drup.2020.100742 33429249

[cit0035] O’Brien MER, Wigler N, Inbar M et al. 2004. Reduced cardiotoxicity and comparable efficacy in a phase IIItrial of pegylated liposomal doxorubicin HCl(CAELYX^TM^/Doxil®) versus conventional doxorubicin forfirst-line treatment of metastatic breast cancer. Ann Oncol. 15:440–449. 10.1093/annonc/mdh097 14998846

[cit0036] O’Shaughnessy J, Emens LA, Chui SY et al. 2021. Patterns and predictors of first-line taxane use in patients with metastatic triple-negative breast cancer in US clinical practice. Curr Oncol. 28:2741–2752. 10.3390/curroncol28040239 34287291 PMC8293053

[cit0037] Prabhakar U, Maeda H, Jain RK et al. 2013. Challenges and key considerations of the enhanced permeability and retention effect for nanomedicine drug delivery in oncology. Cancer Res. 73:2412–2417. 10.1158/0008-5472.CAN-12-4561 23423979 PMC3916009

[cit0038] Rabah SO. 2010. Acute taxol nephrotoxicity: histological and ultrastructural studies of mice kidney parenchyma. Saudi J Biol Sci. 17:105–114. 10.1016/j.sjbs.2010.02.003 23961065 PMC3730725

[cit0039] Schmid P, Rugo HS, Adams S et al. 2020. Atezolizumab plus nab-paclitaxel as first-line treatment for unresectable, locally advanced or metastatic triple-negative breast cancer (IMpassion130): updated efficacy results from a randomised, double-blind, placebo-controlled, phase 3 trial. Lancet Oncol. 21:44–59. 10.1016/S1470-2045(19)30689-8 31786121

[cit0040] Skoczen SL, Snapp KS, Crist RM et al. 2020. Distinguishing pharmacokinetics of marketed nanomedicine formulations using a stable isotope tracer assay. ACS Pharmacol Transl Sci. 3:547–558. 10.1021/acsptsci.0c00011 32566919 PMC7296544

[cit0041] Stetefeld J, McKenna SA, Patel TR. 2016. Dynamic light scattering: a practical guide and applications in biomedical sciences. Biophys Rev. 8:409–427. 10.1007/s12551-016-0218-6 28510011 PMC5425802

[cit0042] Sweet CS, Emmert SE, Seymour AA et al. 1987. Measurement of cardiac output in anesthetized rats by dye dilution using a fiberoptic catheter. J Pharmacol Methods. 17:189–203. 10.1016/0160-5402(87)90050-7 3302543

[cit0043] Teng X, Guan Z, Yao Z et al. 2005. A tolerability study of a cremophor free, nanoparticle albumin bound paclitaxel intravenously administered in Chinese patients with advanced solid tumor. JCO. 23:5571–5571. 10.1200/jco.2005.23.16_suppl.5571 15566651

[cit0044] Wang B, Sun T, Zhao Y et al. 2022. A randomized phase 3 trial of gemcitabine or nab-paclitaxel combined with cisPlatin as first-line treatment in patients with metastatic triple-negative breast cancer. Nat Commun. 13:4025. 10.1038/s41467-022-31704-7 35821019 PMC9276725

[cit0045] Wang H, Li Q, Alam P et al. 2023. Aggregation-induced emission (AIE), life and health. ACS Nano. 17:14347–14405. 10.1021/acsnano.3c03925 37486125 PMC10416578

[cit0046] Xiong G. 2023. Strategies for improving the effectiveness of nanomedicine in triple negative breast cancer model. UCL (University College London).

[cit0047] Xiong G, Li S, Schätzlein AG et al. 2025a. Amphiphilic albumin-based nanoparticles designed for the efficient delivery of taxanes. Int J Pharm. 682:125965. 10.1016/j.ijpharm.2025.125965 40664342

[cit0048] Xiong G, Li S, Schätzlein AG et al. 2025b. Albumin-based nanoparticles encapsulating SN-38 demonstrate superior antitumor efficacy compared to irinotecan. Drug Deliv. 32:2545519. 10.1080/10717544.2025.2545519 40819311 PMC12360046

[cit0049] Xiong G, Schätzlein AG, Uchegbu IF. 2025c. Acetyl-lysine human serum albumin nanoparticles activate CD44 receptors, with preferential uptake by cancer stem cells, leading to tumor eradication. J Control Release. 382:113632. 10.1016/j.jconrel.2025.113632 40139395

[cit0050] Xu X, Wang L, Xu H-Q et al. 2013. Clinical comparison between paclitaxel liposome (Lipusu^®^) and paclitaxel for treatment of patients with metastatic gastric cancer. Asian Pacific J Cancer Prevention. 14:2591–2594. 10.7314/APJCP.2013.14.4.2591 23725180

[cit0051] Xu G, Zhang Q, Cheng R et al. 2025. Survival strategies of cancer cells: the role of macropinocytosis in nutrient acquisition, metabolic reprogramming, and therapeutic targeting. Autophagy. 21:693–718. 10.1080/15548627.2025.2452149 39817564 PMC11925119

[cit0052] Yan D, Wang D, Tang BZ. 2025. In vivo, clinical and translational aspects of aggregation-induced emission. Nat Rev Bioeng. 3:976–991. 10.1038/s44222-025-00342-1

[cit0053] Zhao M, Lei C, Yang Y et al. 2015. Abraxane, the nanoparticle formulation of paclitaxel can induce drug resistance by up-regulation of P-gp. PLoS One. 10:e0131429. 10.1371/journal.pone.0131429 26182353 PMC4504487

[cit0054] Zhao Z, Zhang H, Lam JWY et al. 2020. Aggregation‐induced emission: new vistas at the aggregate level. Angew Chem Int Ed. 59:9888–9907. 10.1002/anie.201916729 32048428

