# Supplementary material for: The colloidal stability of albumin-based drug delivery systems has a profound effect on tumoricidal activity
Source: Drug Deliv. 2026 Jan 20;33(1):2614801. doi: 10.1080/10717544.2026.2614801 (PMC12821350; doi:10.1080/10717544.2026.2614801)
Supplement: Drug delivery supplementary materials GX.docx [file IDRD_A_2614801_SM6434.docx]

**Supplementary materials for**

**The Colloidal Stability of Albumin Based Drug Delivery Systems Has a Profound Effect on Tumoricidal Activity**

*Guojun Xiong^a^, Chengwei Jiang^a^, Andreas G. Schätzlein^a,b^, Ijeoma F. Uchegbu^a,b,c,^**

^a^ School of Pharmacy, University College London, 29-39 Brunswick Square, London, WC1N 1AX, United Kingdom

^b^ Nanomerics Ltd., 6^th^ Floor, 2 London Wall Place, London, EC2Y 5AU, United Kingdom

^c^ Wolfson College, University of Cambridge, Cambridge CB3 9BB

* Corresponding author

Contact: ijeoma.uchegbu@ucl.ac.uk

## Supplementary Table

Table S1. Hydrodynamic diameter and polydispersity index (PDI) of PTX-HSA nanoparticles, Abraxane and HSA-PLA (PTX) in PBS (pH 7.4). Data are presented as mean ± SD, n = 3.

|  | PTX-HSA NPs | Abraxane | HSA-PLA (PTX) |
| --- | --- | --- | --- |
| Hydrodynamic diameter (nm) | 193 ± 30 | 128 ± 8 | 151 ± 9 |
| PDI | 0.12 ± 0.04 | 0.05 ± 0.02 | 0.06 ± 0.02 |

## Supplementary Figure


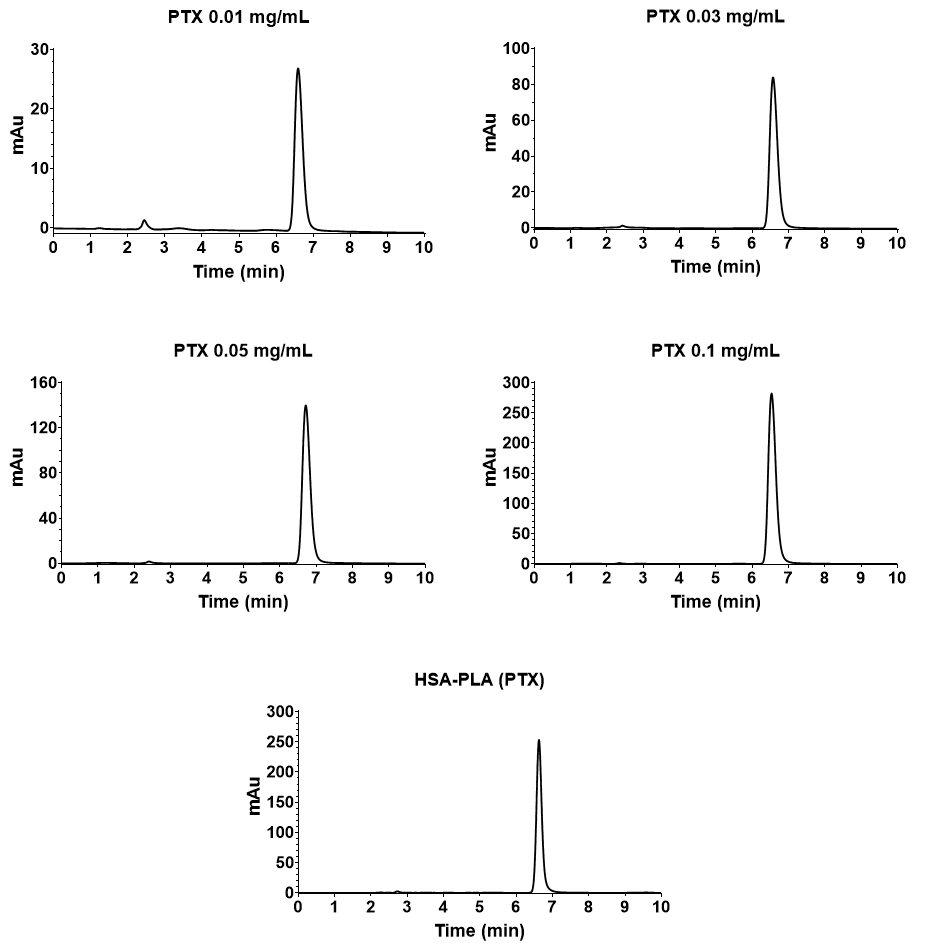


Figure S1. High-Performance Liquid Chromatography (HPLC) chromatograms of PTX standard and HSA-PLA (PTX)

The mobile phase consisted of 60% acetonitrile (HPLC grade) and 40% water (HPLC grade). The stationary phase was a C18 column (4.6 × 150 mm, 5 µm). The flow rate was 0.5 mL/min. Paclitaxel (PTX) was detected at 227 nm with a retention time of approximately 6.5 minutes.


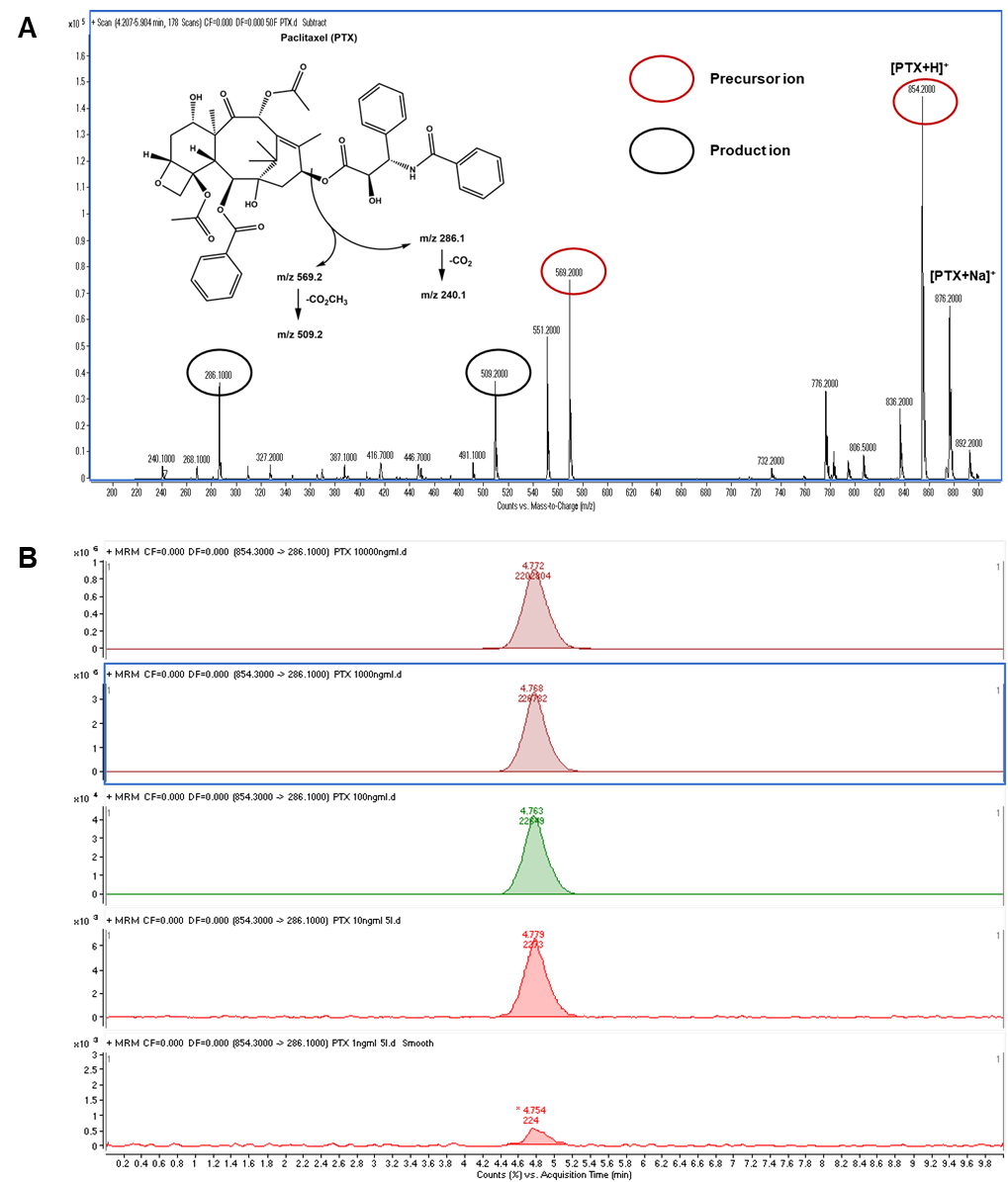


Figure S2. LC-MS/MS calibration curve determination method.

A: MS range-scan mode was used to detect and identify paclitaxel ions within the mass range of 200–900 m/z. The ion at 854 m/z was selected as the precursor ion, and the ion at 286 m/z was chosen as the product ion.

B: LC-MS/MS chromatograms (top to bottom) of paclitaxel at concentrations of 10,000 ng/mL, 1,000 ng/mL, 100 ng/mL, 10 ng/mL, and 1 ng/mL acquired under multiple reaction monitoring (MRM) mode.


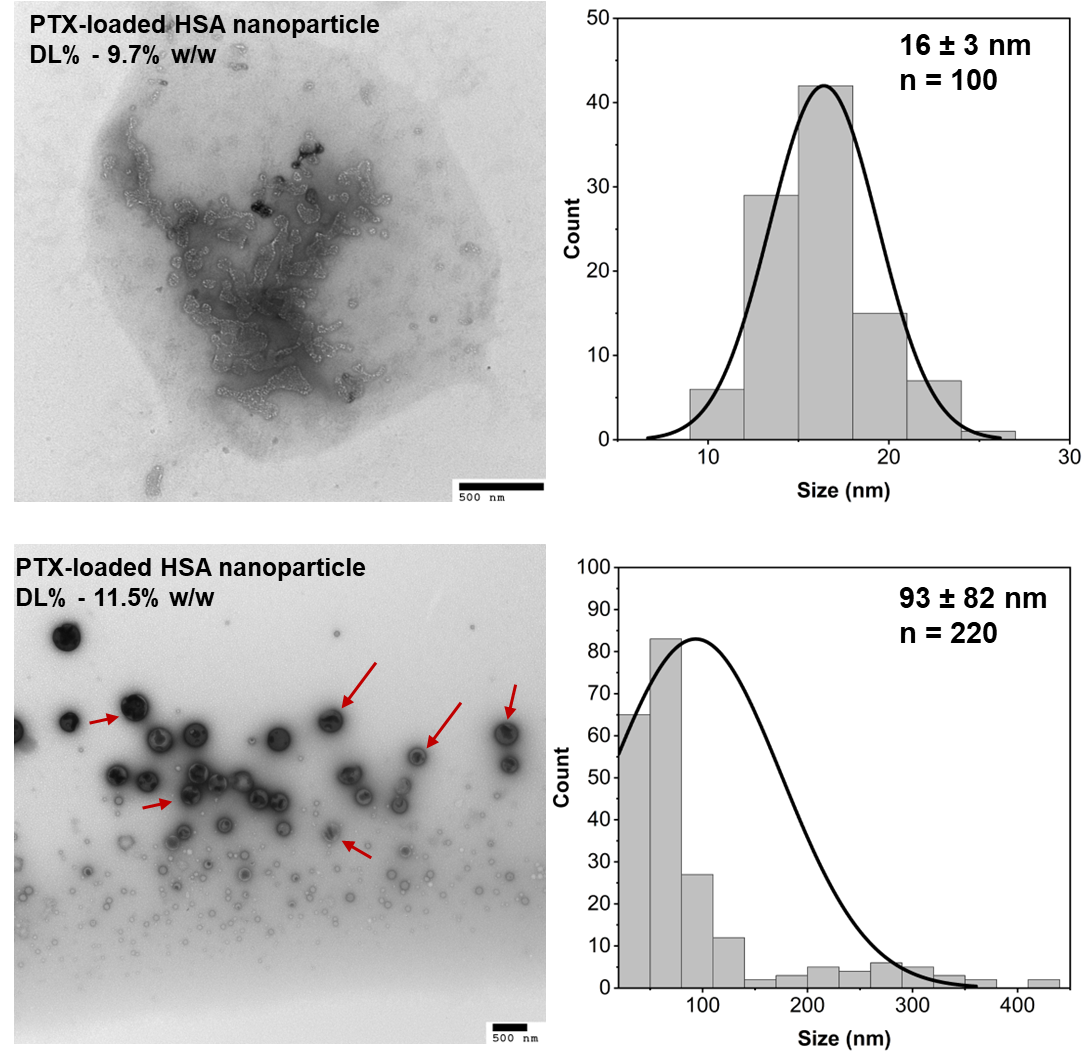


Figure S3. Morphology and size distribution of our prepared PTX-loaded HSA nanoparticles.

(Top) PTX-HSA nanoparticles with a drug loading of 9.7% (w/w) exhibited a mean diameter of 16 ± 3 nm (n = 100).
(Bottom) PTX-HSA nanoparticles with a drug loading of 11.5% (w/w) exhibited a mean diameter of 93 ± 82 nm (n = 220). Cracked PTX-HSA nanoparticles are indicated by red arrows.


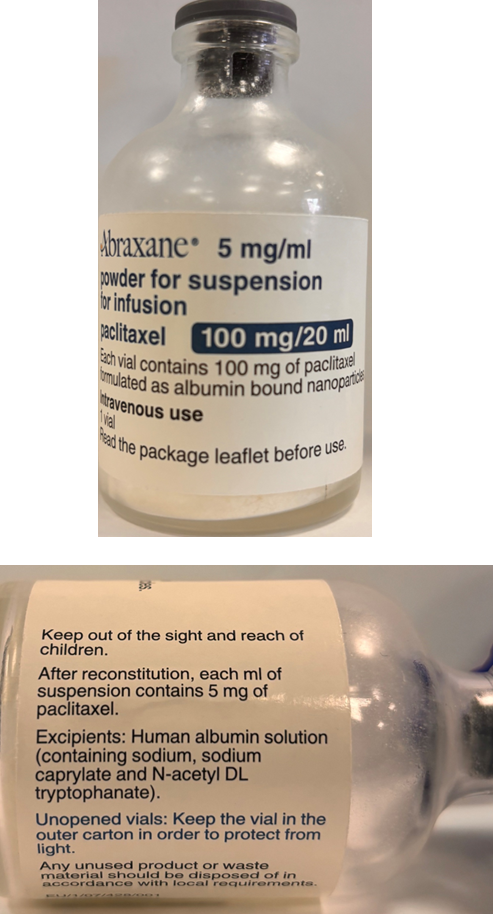


Figure S4. Photographs of the Abraxane vial and its label information.


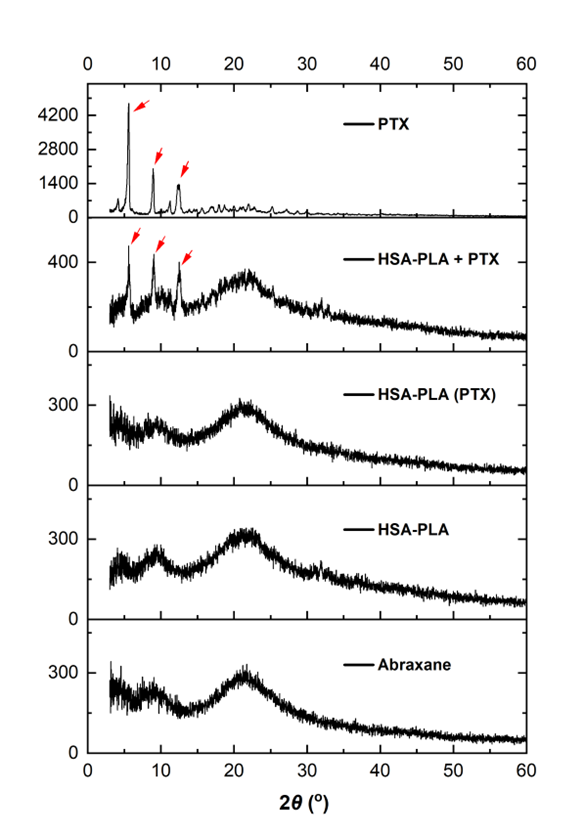


**Figure S5. XRD patterns of PTX standard, physical mixture of HSA-PLA and PTX, PTX-loaded HSA-PLA nanoparticles, HSA-PLA blank nanoparticles and Abraxane.**

Adapted from our previous open access publications (*Amphiphilic albumin-based nanoparticles designed for the efficient delivery of taxanes*; *Acetyl-lysine human serum albumin nanoparticles activate CD44 receptors, with preferential uptake by cancer stem cells, leading to tumor eradication*), which are distributed under the terms of the Creative Commons Attribution (CC BY) license.


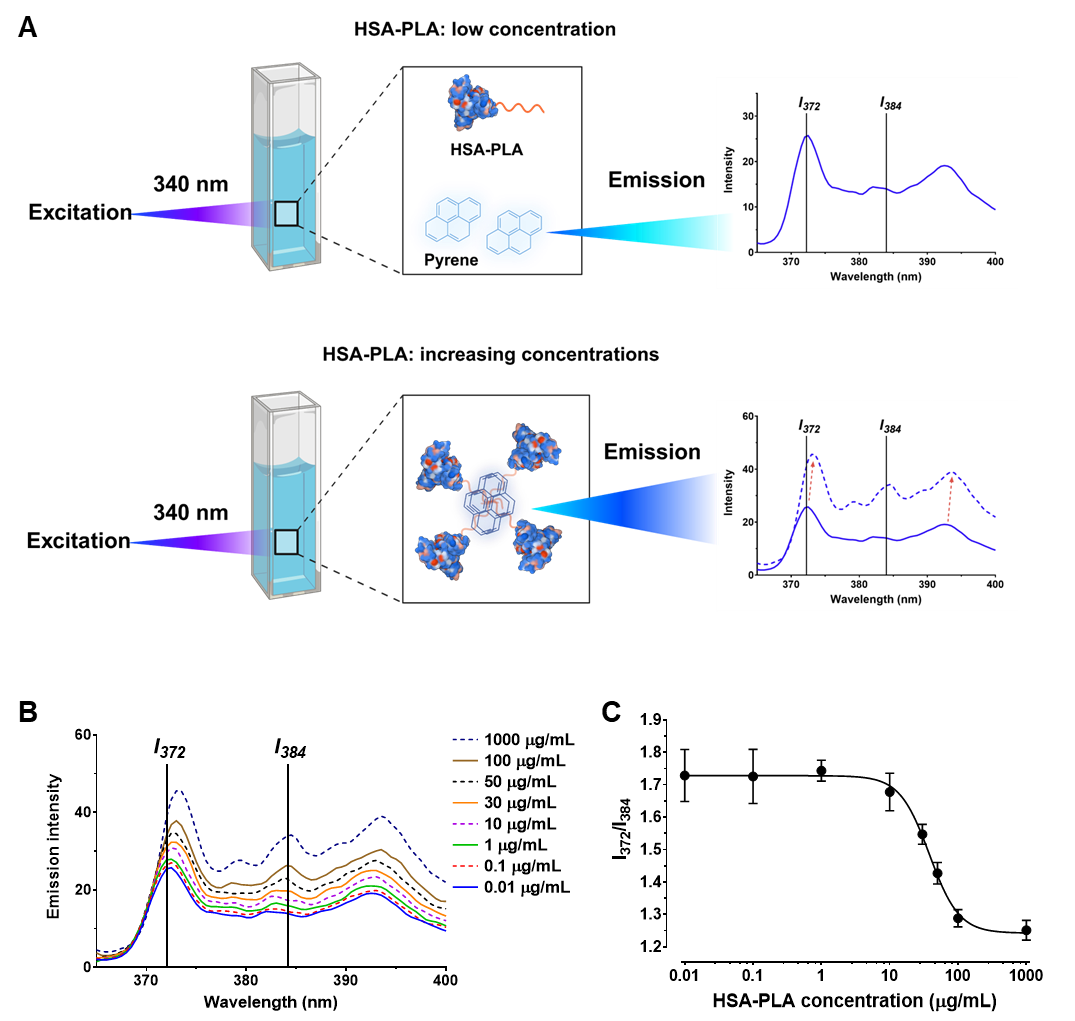


**Figure S6. Determination of the self-assembly and critical micelle concentration (CMC) of HSA–PLA nanoparticles using pyrene fluorescence.**

(A) Schematic illustration showing free pyrene molecules in solution were excited at a wavelength of 340 nm and exhibited characteristic fluorescence emission peaks. When pyrene molecules were encapsulated within and stacked in the hydrophobic core of nanoparticles, their emission showed a slight red shift and enhanced intensity, even at the same pyrene concentration.

(B) Representative fluorescence spectra of pyrene in the presence of different concentrations of HSA-PLA nanoparticles, showing changes in I₃₇₂/I₃₈₄ ratio as the nanoparticle concentration increases.
(C) The I₃₇₂/I₃₈₄ ratio plotted against logarithmic concentrations of HSA–PLA nanoparticles. The CMC was determined from the inflection point of the Boltzmann-sigmoidal fitting curve.

Figure reproduced from Xiong et al., “Amphiphilic albumin-based nanoparticles designed for the efficient delivery of taxanes,” International Journal of Pharmaceutics, 2025, 125965 (open access), with permission under the journal’s CC BY 4.0 license.


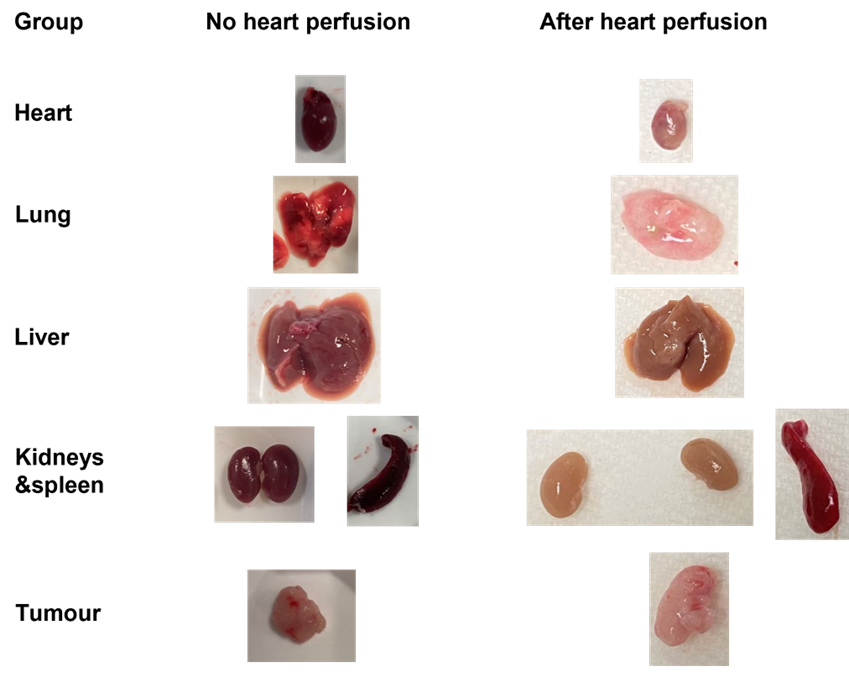


Figure S7. Visual comparison of mouse organs with and without heart perfusion. The visual change is due to the removal of blood from organs following heart perfusion. This figure is used to demonstrate the necessity of performing heart perfusion prior to analyzing paclitaxel levels in organs, by minimizing the influence of paclitaxel in the blood.


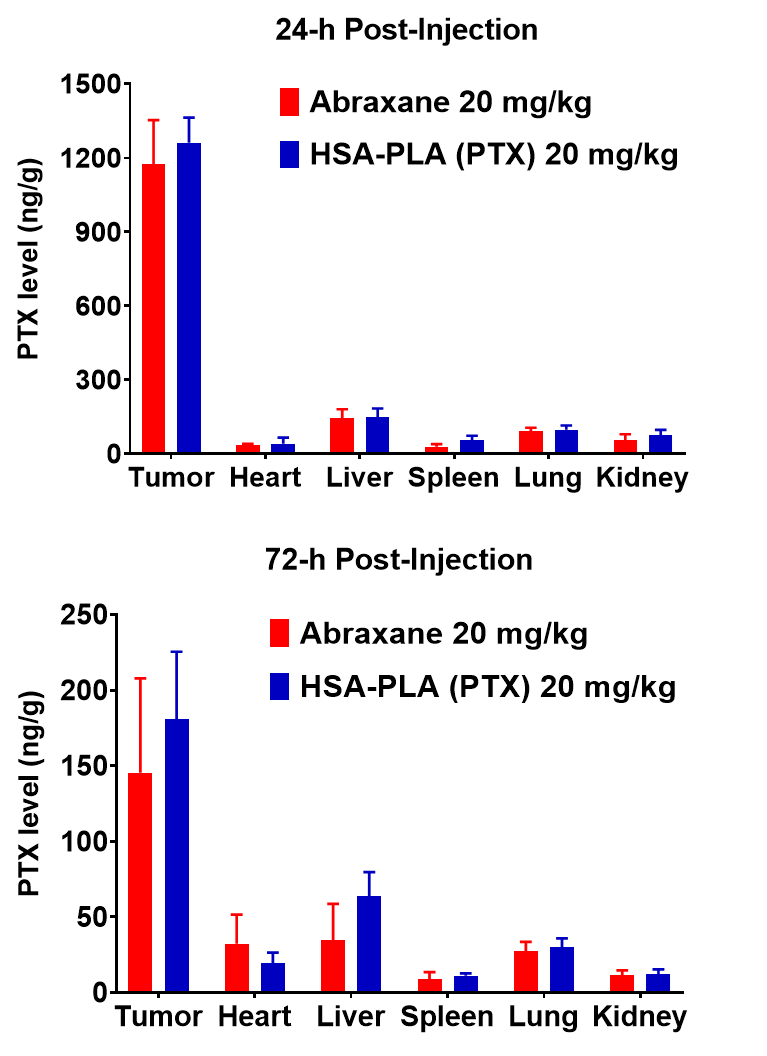


Figure S8. Biodistribution of PTX in major organs and tumors 24 and 72 hours after intravenous injection of Abraxane or HSA-PLA (PTX) in 4T1 tumor-bearing mice.

Data are presented as mean ± SD (n = 3 per group).
